# Perspectives on the utilization of *Aegilops* species containing the U genome in wheat breeding: a review

**DOI:** 10.3389/fpls.2025.1661257

**Published:** 2025-10-27

**Authors:** Ekaterina D. Badaeva, Maida Jazmin González Franco, Olga Razumova, Nadezhda A. Tereshchenko, Mikhail Divashuk

**Affiliations:** ^1^ Laboratory of Applied Genomics and Crop Breeding, All-Russian Research Institute of Agricultural Biotechnology, Russian Academy of Agricultural Sciences, Moscow, Russia; ^2^ Laboratory of Genetic Basis of Plant Identification, Vavilov Institute of General Genetics, Russian Academy of Sciences, Moscow, Russia

**Keywords:** wheat, *Aegilops*, u-genome, interspecific hybridization, alien introgression, agriculturally valuable traits

## Abstract

The genus *Aegilops* L. is the closest wild relative of wheat (*Triticum* L.), which contributed two of the three genomes to cultivated wheat. The genus *Aegilops* comprises 23 species differing in ploidy level and genome composition; diploid species possess the C, D, M, N, S, and U genome types, whereas various genome combinations are identified in tetraploid and hexaploid forms. The U genome is present in diploid *Ae. umbellulata* and eight polyploid species [*Ae. triuncialis*, *Ae. biuncialis*, *Ae. geniculata*, *Ae. peregrina*, *Ae. kotschyi*, *Ae. columnaris*, *Ae. neglecta* (4× and 6×), and *Ae. juvenalis*]. Some of these species have a wide distribution range, resulting in high adaptive capacity to various environmental conditions, and can serve as a valuable source of genetic diversity and useful genes for wheat breeding. The U genome is substantially rearranged relative to the genomes of common wheat, which hampers the direct transfer of useful traits from *Aegilops* to wheat. However, many genes conferring resistance to leaf rust (*Lr9*, *Lr76*, *Lr57*, *Lr54*, *Lr59*, *Lr58*), stripe rust (*Yr70*, *Yr40*, *Yr37*, *Yr42*), stem rust (*Sr53*), nematodes (*CreX*, *CreY*, *Cre7*), and various abiotic stresses have been successfully introgressed from *Aegilops* into the wheat genome. In this review, we describe the status of the contribution of *Aegilops* species carrying the U genome to wheat improvement, the methods used by different scientific teams to transfer genetic material, and the future prospective of exploitation of their useful traits in practical breeding.

## Introduction

The genus *Aegilops* L. (family Poaceae) comprises 23 annual herbaceous species (Van Slageren, 1994; Kilian et al., 2011). Most of them are native to the Middle East and the Mediterranean, but several species also grow in other regions, such as northwestern Europe, Ukraine, Crimea, the Caucasus, north-Saharan Africa, Central Asia, and western China (Zhukovsky, 1928; Eig, 1929; Hammer, 1980; Boguslavsky and Golik, 2003; Kilian et al., 2011; Feldman and Levy, 2023). Several allotetraploid species have been introduced into the United States, of which *Ae. cylindrica* is now becoming widespread (Gandhi et al., 2009). The greatest diversity of *Aegilops* species is concentrated in the central part of the genus’ range, in the Fertile Crescent (Zhukovsky, 1928; Hammer, 1980; Van Slageren, 1994; Kilian et al., 2011). In such countries as Iran, Iraq, Syria, and Turkey, up to 13–17 of the 23 *Aegilops* species occur, while at the periphery of the range, e.g., in Hungary and Ukraine (eastern Europe), Kazakhstan (Middle Asia), or China and India (South-Eastern Asia), only 2–3 species are recorded (Zhukovsky, 1928).

*Aegilops* are classified into diploid (2*n*=2*x*=14), tetraploid (2*n*=2*x*=28), and hexaploid (2*n*=2*x*=42) species; at all ploidy levels, the species differ in genomic composition (Kihara, 1954; Hammer, 1980; Badaeva et al., 1996; Dvořák, 1998; Badaeva et al., 2002; Badaeva et al., 2021). Six main types of diploid nuclear genomes are currently distinguished among *Aegilops* species, in particular, S (the Sitopsis section), D (*Ae. tauschii*), M (*Ae. comosa*), N (*Ae. uniaristata*), U (*Ae. umbellulata*), and C (*Ae. caudata*) (Kihara, 1954; Kimber and Tsunewaki, 1988; Badaeva et al., 1996; Dvořák, 1998; Kilian et al., 2011; Sharma et al., 2021). The genome of *Amblyopyrum muticum*, which some taxonomists also classify as a member of the genus *Aegilops* (Zhukovsky, 1928; Ohta and Saruhashi, 1999), is designated by the symbol T (Hammer, 1980; Dvořák, 1998; Edet et al., 2018) (**Table 1**). Recent studies showed that the S genome of *Ae.* sp*eltoides* differs significantly from the S* genomes of other *Sitopsis* species (Dvořák, 1998; Ruban and Badaeva, 2018; Avni et al., 2022; Li et al., 2022) and is phylogenetically close to the T genome of *A. mutica* (Ohta and Saruhashi, 1999; Edet et al., 2018; Parisoid and Badaeva, 2020; Avni et al., 2022; Adhikari et al., 2023). Some diploid genomes, like D, S, and T, are not modified relative to the ancestral, and some modifications are found in the M genome, whereas U, C, and N genomes underwent significant structural rearrangements due to species-specific translocations and inversions (Iqbal et al., 2000; Danilova et al., 2017; Parisoid and Badaeva, 2020; Li et al., 2021; Said et al., 2021; Abrouk et al., 2023; Li et al., 2024; Singh et al., 2024).

**Table 1:** Characteristics of genomes of *Aegilops* species.

Species name^1^	Ploidy level	Nuclear genome size (1C = Mbp)^2^	Nuclear genome^3^	Plasmon type^4^	Chloroplast (cp) genome^4^	Mitochondrial (mt) genome^4^	Synonyms
Section Sitopsis							
*Ae.* sp*eltoides* Tausch	2×	4,998.00	S	S	S, G	S, G, G′	*Aegilops auscheri* Boiss.; *Ae. ligustica* (Savign.) Coss.; *Triticum* sp*eltoides* (Tausch) Å. Löve
*Ae. bicornis* (Forssk.) Jaub. & Spach	2×	6,958.00	S^b^	S^b^	S^b^	S^b^	*Triticum bicorne* Forssk.
*Ae. searsii* Feldman & Kislev ex Hammer	2×	5,782.00	S^s^	S^v^	S^v^	S^v^	*Triticum searsii* Feldman & Kislev
*Ae. sharonensis* Eig.	2×	6,958.00	S^sh^	S^l^	S^l^	S^l^	*Triticum sharonense* (Eig.) Feldman & Sears; *Ae. longissima* ssp. *sharonensis*
*Ae. longissima* Schweinf. & Muschl.	2×	5,978.00	S^l^	S^l^	S^l′^	S^l′^	*Triticum longissimum* (Schweinf. & Muschl.) Bowden
Section Comopyrum							
*Ae. comosa* Sm. in Sibth. & Sm subsp. *comosa*	2×	6,076.00	M	M	M	M	*Triticum comosum* (Sm. in Sibth. & Sm.) K. Richt.
*Ae. comosa* subsp. *heldreichii*	2×	N/A	M^h^	M^h^	M^h^	M^h^	*Ae heldreichii* (Holzm. ex Boiss.) Halácsy
*Ae. uniaristata* Vis.	2×	6,174.00	N	N	N	N	*Triticum uniaristatum* (Vis.) K. Richt.
Section Vertebrata							
*Ae. tauschii* Coss.	2×	4,998.00	D	D	D	D	*Aegilops squarrosa* L.; *Triticum tauschii* (Coss.) Schmalh.
*Ae. ventricosa* Tausch	4×	9,604.00	DN	D	D	D	*Triticum ventricosum* (Tausch) Ces., Pass & Gibelli
*Ae. crassa* Boiss.	4×	10,290.00	D^1^X^cr^	D^2^	D^2^	D^2^	*Triticum crassum* (Boiss.) Aitch. & Hemsl., 4×
*Ae. crassa* Boiss.	6×	15,386.00	D^1^X^cr^D^2^	D^2^	D^2^	D^2^	*Triticum crassum* (Boiss.) Aitch. & Hemsl., 6×; *Aegilops trivialis* (Zhuk.) Migusch. et Chak.
*Ae. vavilovii* (Zhuk.) Chennav.	6×	17,934.00	D^1^X^cr^S^s^	D^2^	D^2^	D^2^	*Ae. crassa* var. palaestina Eig.; *Triticum syriacum* Bowden
*Ae. juvenalis* (Thell.) Eig	6×	18,424.00	D^1^X^cr^U^j^	D^2^	D^2^	D^2^	*Trriticum juvenale* Theil.; *T. turcomanicum* (Rosh.) Bowden; *Ae. turcomanica* Rosh.
Section Cylindropyron							
*Ae. caudata* L.	2×	4,508.00	C	C	C	C	*Triticum caudatum* (L.) Godr. & Gren.; *Triticum dichasians* Bowden; *Aegilops dichasians* (Bowden) Humphries; *Aegilops markgrafii* (Greuter) Hammer
*Ae. cylindrica*	4×	9,594.00^5^	DC; CD	D′	D′	D	*Triticum cylindricum* (Host.) Ces. Pass & Gibelli
Section Aegilops							
*Ae. umbellulata* Zhuk.	2×	4,998.00	U	U	U	U	*Triticum umbellulatum* (Zhuk.) Bowden
*Ae. triuncialis* L.	4×	9,647.90^5^	UC; CU	U; C	U; C	U; C	*Triticum triunciale* (L.) Raspail; *Ae. persica* Boiss
*Ae. peregrina* (Hackel) Maire et Weiller	4×	12,269.60	S^l^U	S^v^	S^v^	S^v^	*Triticum peregrinum* Hack.; *Ae. variabilis* Eig.
*Ae. columnaris* Zhuk.	4×	10,290.00	UX^c^	U; U^2^	U’	U’	*Triticum columnare* (Zhuk.) Morris & Sears
*Ae. kotschyi* Boiss.	4×	12,279.63^5^	S^l^U	S^v^	S^v^	S^v^	*Triticum kotschyi* (Boiss.) Bowden
*Ae. geniculata* Roth.	4×	10,084.20	UM°	M°	M°	U′, M°	*Triticum ovatum* (L.) Raspail.; *Ae. ovata* L.
*Ae. biuncialis* Vis.	4×	11,074.00	UM^b^	U	U	U	*T. biunciale* (Richrt.); *T. macrochaetum* (Shuttl. & Huet.) Richt.; *Ae. lorentii* Hochst.
*Ae. neglecta* Req. ex Bertol.	4×	10,427.20	UX^t^	U	U	U	*Triticum neglectum* (Req. ex Bertol.) *Greuter*; *T. triaristatum* (Willd.) Godr. & Gren.; *Ae. triaristata* (4×) Willd.
*Ae. recta* (Zhuk.) Chennav.	6×	18,179.00	UX^t^N	U	U	U	*Triticum rectum* (Zhuk,) Bowden; *T. triaristatum* (6×) (Willd.) Godr. & Gren.; *Ae. neglecta* Req. Ex Bertol (6×)
Section Amblyopyrum							
*Amblyopyrum muticum* (Boiss.) Eig.	2×	6,174.00	T	T, T^2^	T, T^2^	T, T^2^	*Aegilops mutica* Boiss.; *Aegilops tripsacoides* Jaub. & Spach., *Triticum tripsacoides* Bowden *Triticum muticum* (Boiss.) Hack.

^1^Species names are given in accordance with Van Slageren (1994) and Sharma et al (Sharma et al., 2021).

^2^Nuclear DNA content is taken from the C-value database (https://cvalues.science.kew.org/).

^3^Nuclear genome is designated according to Dvořák (1998).

^4^Plasmon type, chloroplast (Cp), and mitochondrial (Mt) genomes are designated according to Tsunewaki (2009).

^5^Nuclear DNA content according to Eilam et al (Eilam et al., 2010).

**Figure 1:**
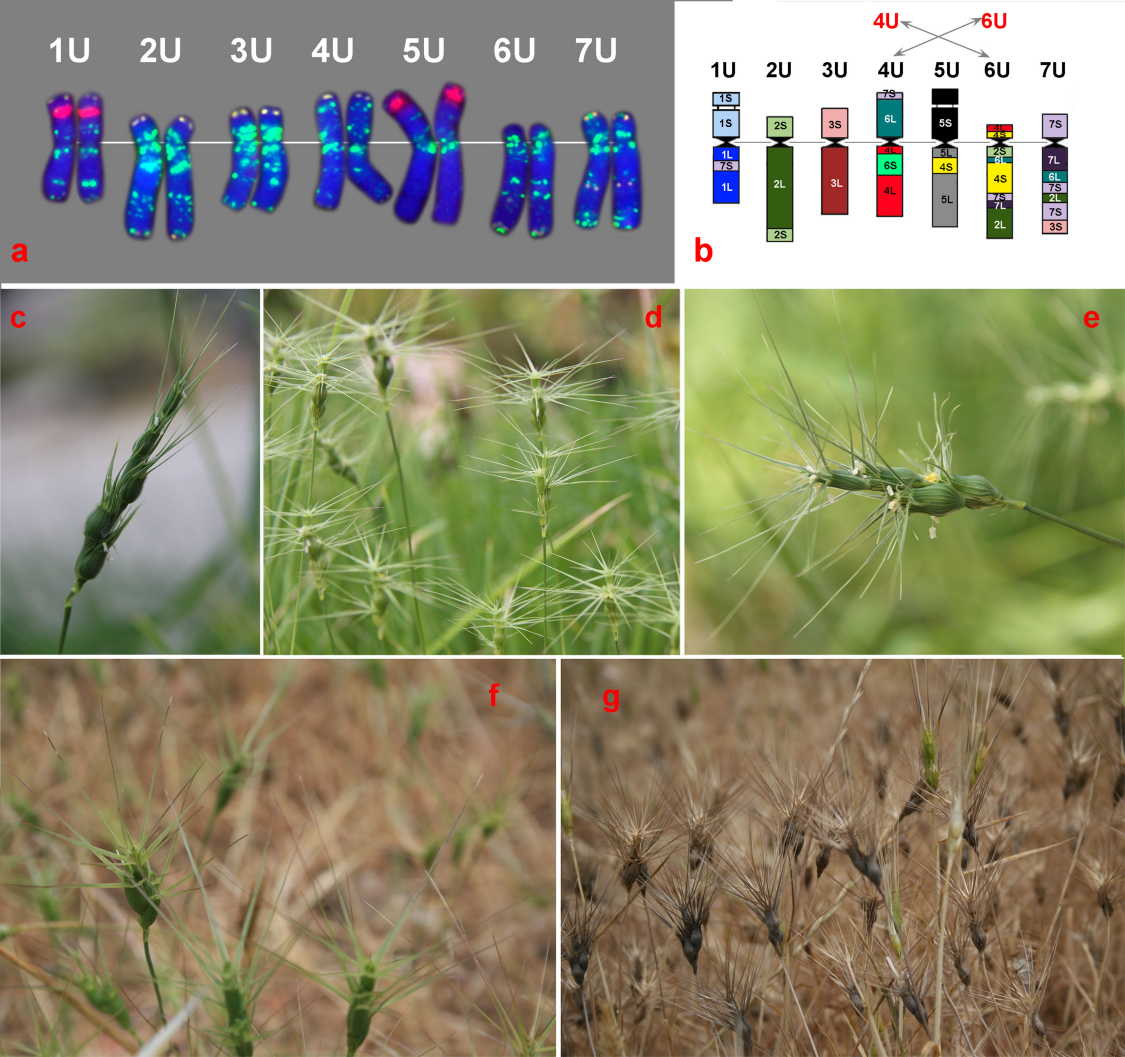
Chromosome organization of the U genome **(a, b)** and morphological variation of diploid *Aegilops umbellulata* **(d)** and some polyploid species of the U genome group: **(c)** *Ae. juvenalis*, **(e)** *Ae. columnaris*, **(f)** *Ae. peregrina*, and **(g)** *Ae. geniculata.* **(a)** Distribution of GAA_n_ (green), pTa71 (red), and pSc119.2 (yellow) probes on *Ae. umbellulata* (AE 822) visualized by FISH; **(b)** the structural rearrangements of the U genome chromosomes [modified after Said et al (Said et al., 2021)].

The U genome of *Ae. umbellulata* is one of the best characterized genomes. Nearly complete sets of wheat-*Ae. umbellulata* chromosome addition, substitution, and translocation lines have been produced (Schneider et al., 2008). The homoeologous relationships of *Ae*. *umbellulata* chromosomes were established based on meiotic chromosome pairing behavior and compensation ability in substitution lines (Chapman and Riley, 1970; Athwal and Kimber, 1972; Riley et al., 1973; Chapman et al., 1975; Koebner and Shepherd, 1986; Reader and Miller, 1987), analysis of storage protein (Shepherd, 1973; Brown et al., 1979; Lawrence and Shepherd, 1981) and isozymes (Benito et al., 1987), and chromosome painting with bulked group-specific oligo-probes (Li et al., 2021). The species karyotype was constructed, and the intraspecific chromosome diversity of *Ae. umbellulata* was assessed by C-banding (Friebe et al., 1995) and FISH with various DNA probes (Badaeva et al., 1996; Molnár et al., 2011; Song et al., 2020). The U genome chromosomes were found to be highly asymmetric due to species-specific translocations (**Figure 1A**). Thus, according to RFLP analysis of wheat-*Ae. umbellulata* addition lines, the U genome diverged as a result of 11 translocations and inversions involving all seven chromosomes (Zhang et al., 1998). Results of physical mapping of cDNA probes and comparative single-gene FISH analysis confirmed these translocations and showed that 4U and 6U are the most rearranged chromosomes of *Ae. umbellulata* (**Figure 1B**). According to the authors’ data, four out of the seven group 4-specific cDNA probes were mapped on 6U and all the six group 6-specific probes—on 4U pointing to multiple reciprocal translocations between 4U and 6U. Based on these data, Zhang et al. (1998) and Said et al. (2021) suggested to correct the genetic classification of *Ae. umbellulata* chromosome by re-placing 4U and 6U. Another species-specific translocation involved chromosomes 3U and 7U (Said et al., 2021).

Sequencing and chromosome-scale assembly of *Ae. umbellulata*, acc. TA1851 (Abrouk et al., 2023), as well as high-quality near telomere-to-telomere genome assembly (acc. PI 554389 (Singh et al., 2024), confirmed significant structural reorganization of the U genome. As in other *Aegilops* species, the major part of the U genomes was found to be composed of mobile elements, particularly retrotransposons. Genome assembly of PI 554389 allowed annotation of 78,076 gene models, 48,366 of which were high-confidence (HC) genes. A total of 2,162 among the HC genes were resistance gene analogs (RGAs) (Singh et al., 2024). This finding implies the great potential of *Ae. umbellulata* as a source of resistance genes for wheat improvement.

**Figure 2:**
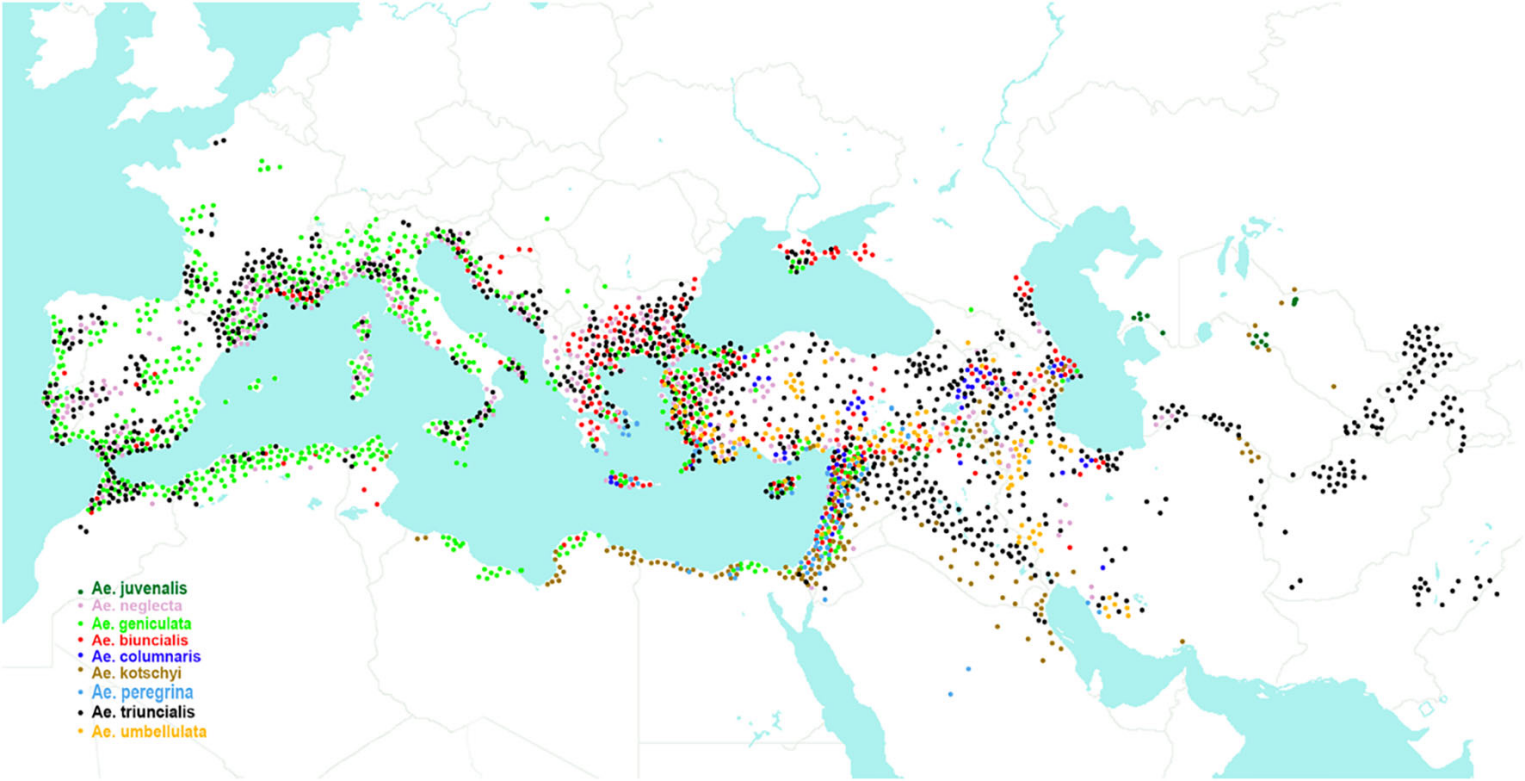
Distribution area of *Aegilops* species containing the U genome.

Polyploid *Aegilops* species have different genome compositions (Kihara, 1954; Hammer, 1980; Kimber and Tsunewaki, 1988; Dvořák, 1998; Badaeva et al., 2002; Kilian et al., 2011; Badaeva et al., 2021). Based on the presence of the “pivotal” genome, Kimber and Feldman (1987) divided them into two main clusters: the U genome cluster and the D genome cluster. The U genome cluster includes eight species: tetraploid *Ae. triuncialis*, *Ae. kotschyi*, *Ae. peregrina*, *Ae. biuncialis*, *Ae. geniculata*, *Ae. columnaris*, *Ae. neglecta*, 4x, and hexaploid *Ae. neglecta* subsp. *recta* (thereafter *Ae. recta*) (**Table 1**). In addition, the U genome is found in hexaploid *Ae. juvenalis*, belonging to the D genome cluster (Kihara et al., 1959; Kimber and Feldman, 1987; Dubkovsky and Dvořák, 1995). Analysis of *Ae. juvenalis* using C-banding and FISH revealed significant rearrangements of the U^j^ genome chromosomes relative to *Ae. umbellulata* (Badaeva et al., 2021). Most species from the U genome cluster grow in the Mediterranean and the Middle East, while *Ae. juvenalis* occurs mainly in the Middle and Central Asia (**Figure 2**).

The cytoplasmic genome (plasmon) of five species of this group is identical or slightly modified relative to the cytoplasmic genome of *Ae. umbellulata*, indicating that it was their maternal parent and cytoplasm donor (**Table 1**). The plasmon of *Ae. kotschyi* and *Ae. peregrina* corresponds to the cytoplasmic genome of *Ae. searsii*, while the plasmon of *Ae. juvenalis* is identical to that of *Ae. crassa* (Tsunewaki, 2009). The origin of *Ae. geniculata* plasmon is not clearly understood, since it differs significantly from other *Aegilops* species. A proposed hypothesis suggests its inheritance from *Ae. mutica* (Maan, 1977; Tsunewaki, 2009).

Phylogenetically, the genus *Aegilops* is closely related to the genus *Triticum* and played a major role in the evolution of wheat (Levy and Feldman, 2022). Hexaploid bread wheat *Triticum aestivum* and tetraploid durum wheat *T. durum* share two common genomes, A and B, which are supplemented by the D genome in hexaploid wheat (Kihara, 1944; McFadden and Sears, 1946; Lilienfeld, 1951; Dvořák et al., 1998; El Baidouri et al., 2017; Levy and Feldman, 2022). The A genome of *T. durum* and *T. aestivum* is most similar to the A^u^ genome of diploid wild wheat *T. urartu* (Dvořák et al., 1988; Dvořák, 1998; Badaeva et al., 2015). The immediate donor of the B genome is not exactly determined, but it is thought to derive from currently extinct species related to *Ae.* sp*eltoides* (Badaeva et al., 1996; Petersen et al., 2006; Salse et al., 2008; Marcussen et al., 2014; Avni et al., 2022; Levy and Feldman, 2022; Adhikari et al., 2023). The donor of the D genome of hexaploid wheat was *Ae. tauschii* (Kihara, 1944; McFadden and Sears, 1946; Lelley et al., 2000; Mizuno et al., 2010; Luo et al., 2017; Singh et al., 2019). Thus, the species from the genus *Aegilops* contributed two of the three genomes to bread wheat (Kihara, 1954; Hammer, 1980; Marcussen et al., 2014; Sharma et al., 2021).

## Methods for transferring genetic material from *Aegilops* species to bread wheat

*Aegilops* species are a valuable genetic resource for expanding the genetic diversity of bread and durum wheat (Feldman and Sears, 1981; Gill et al., 1985; Boguslavsky and Golik, 2003; Colmer et al., 2006; Mujeeb-Kazi et al., 2009; Imadi and Gul, 2019; Kishii, 2019; Kumar et al., 2019; Sharma et al., 2021). Many species of this genus, including *Ae. umbellulata* and the U genome polyploids, exhibit broad morphological (**Figures 1C–G**) and genetic diversity (Dhaliwal et al., 1991; Damania et al., 1992; Monneveux et al., 2000; Zaharieva et al., 2001; Boguslavsky and Golik, 2003; Zaharieva et al., 2003; Molnár et al., 2004; Smith et al., 2004; Ahmadpoor et al., 2014; Ivanizs et al., 2019; Singh et al., 2024), driven by adaptation to diverse eco-geographical conditions (Van Slageren, 1994; Zaharieva et al., 2001; Kilian et al., 2011). Consequently, individual species may contain accessions with contrasting expression of traits, such as drought and heat tolerance (Molnár et al., 2004) and rust resistance (Gill et al., 1985; Valkoun et al., 1985).

Many *Aegilops* species, including the U genome group, do not share common genomes with bread wheat and, according to Harlan and de Wet (1971), belong to the secondary or even tertiary gene pool. Direct transfer of genetic material from such forms to bread wheat is challenging due to the non-crossability of the species and hybrid lethality. Significant modification of the U genome relative to wheat genomes (Zhang et al., 1998; Said et al., 2021; Abrouk et al., 2023; Singh et al., 2024) is an additional constraint restricting its direct utilization in wheat breeding, because structural rearrangements of homoeologous chromosomes decrease their compensation ability and pairing capacity (Zhang et al., 2015; Laugerotte et al., 2022).

A standard scheme for transferring genetic material from species of the secondary and tertiary gene pool, including *Aegilops*, involves the following steps. First, identification of potential donors of target traits by screening accessions from diverse geographic regions. The selected *Aegilops* accession is then crossed to bread wheat to produce F_1_ hybrids. The embryos of such hybrids are mainly weak and underdeveloped and often lack the endosperm (Bai et al., 1994). To overcome these problems and recover viable plants, the embryo rescue technique has been developed (Laibach, 1925; Sharma et al., 1996; Rogo et al., 2023). Chromosome doubling in F_1_ plants is usually induced by colchicine treatment (Blakeslee and Avery, 1937), though spontaneous doubling may also occur through fusion of unreduced gametes or autonomous genome duplication in an allohaploid hybrid (Pyralov; Matsuoka et al., 2014; Fakhri et al., 2016). Alien addition and substitution lines are produced by backcrossing the resulting amphidiploid by bread wheat (Feldman and Sears, 1981). Due to the tendency toward elimination of alien chromosomes over successive generations of interspecific hybrids (Blüthner et al., 1988; Bai et al., 1994; Zhang et al., 2015; Badaeva et al., 2024), it is necessary to monitor the inheritance of *Aegilops* chromosomes using morphological, molecular, or cytological markers.

Since alien chromosomes, in addition to target genes, contain linked loci coding undesirable traits (“linkage drag”), geneticists and breeders are aiming to reduce the size of the introgressed chromosome fragment by inducing wheat–alien translocations (Knott and Dvořák, 1976; Qi et al., 2007). Normally, homoeologous wheat and alien chromosomes do not pair in meiosis (Riley et al., 1959; Naranjo and Benavente, 2015; Perničková et al., 2019; Laugerotte et al., 2022); thus, methods such as irradiation, gametocidal (Gc) factors, genes-promoters of homoeologous pairing, or *Ph1* (*Pairing homoeologous 1—*the key gene regulating meiotic pairing in wheat) suppressors are employed to induce the translocations (Qi et al., 2007; Zhang et al., 2015).

Irradiation and gametocidal factors cause random chromosome breaks, and fusion of the resulting fragments leads to the formation of translocations (Tsujimoto and Tsunewaki, 1985; Chen et al., 2009; Wang et al., 2012; Niranjana, 2017; Said et al., 2024). Most translocations will be non-compensatory, because they occur between non-homoeologous chromosomes (Knott and Dvořák, 1976; Qi et al., 2007; Rather et al., 2017). Rearrangements induced by X-rays cause a reduction of fertility and genomic stability in consecutive generations of the amphiploid and introgressive lines (Molnár et al., 2009; Wang et al., 2012; Wang et al., 2019). On the other hand, irradiation is effective in inducing translocations between chromosomes that do not pair in meiosis, in particular, wheat–alien translocations, since the presence of even a small translocated fragment at the end of a chromosome blocks its recombination with the normal homologous chromosome (Sears, 1976; Jiang et al., 1993; Laugerotte et al., 2022). Irradiation and a gametocidal system allow breaking the linkage between tightly linked loci and thus promote translocation of very small alien chromosome fragments (Masoudi-Nejad et al., 2002); irradiation is also effective in breaking the linkage between target and gametocidal genes (Ragini et al., 2024). Due to a random occurrence of irradiation-induced chromosomal breaks and rejoining, only a small fraction of translocations obtained by these methods found application in breeding (Friebe et al., 1996a; Qi et al., 2007; Kishii, 2019).

Several systems have been developed to produce wheat–alien translocations by inducing pairing and recombination of homoeologous chromosomes. These rely on either suppressing the *Ph1*, which regulates meiotic chromosome pairing in wheat, or introducing genes—promoters of homoeologous pairing from other species (Qi et al., 2007).

The system employing the recessive mutant of common wheat Chinese Spring (thereafter CS), designated CS *ph1b*, is most widely used for inducing wheat–alien translocations via recombination of homoeologous chromosomes. This mutant was obtained by E.R. Sears by irradiating immature pollen (before the onset of meiosis) of Chinese Spring wheat with subsequent pollination of the genetically marked CS line monosomic for 5B chromosome (Sears, 1977). Cytological (Gill and Gill, 1991) and molecular marker analyses (Gyawali et al., 2019) revealed that the *ph1b* mutation is caused by the deletion of a fragment of chromosome 5BL carrying the *Ph1* gene. Another suppressor gene, *Ph2*, with a less pronounced effect on chromosome pairing, was mapped on 3DS (Mello-Sampayo, 1971; Sears, 1976). Recombination between wheat and *Aegilops* chromosomes can also be induced by using CS lines nullisomic or monosomic for chromosome 5B (Sears and Okamato, 1958; Riley and Kempanna, 1963; Koebner and Shepherd, 1986), as well as by the replacement of chromosome 5B by 5RL of rye in substitution or translocation lines (Bielig and Driscoll, 1970; Merker, 1992); however, none of these methods has been used as widely as CS *ph1b.* A temperature-sensitive mutation, *ph1c*, was later produced in durum wheat cultivar Cappelli using irradiation (Dvořák et al., 1984).

The frequency of recombination between wheat and *Aegilops* chromosomes can be increased by gene promoters of homoeologous pairing introduced into the wheat genome from other species. Such genes were found in *Ae.* sp*eltoides* (Dvořák et al., 2006; Li et al., 2017), *Ae. peregrina* (Farooq et al., 1990), *Ae. umbellulata* (Riley et al., 1973), *Ae. geniculata* (Koo et al., 2020), and *Thinopyrum elongatum* (Dvořák, 1987). Two dominant genes suppressing *Ph1—*the *Su1-Ph1* and *Su2-Ph1*—were identified in *Ae.* sp*eltoides* and mapped on chromosomes 3S and 7S, respectively (Dvořák et al., 2006; Li et al., 2017). Chen et al. (1994) transferred the *Su-Ph1* to the long arm of chromosome 4D of CS; the translocated chromosome was able to suppress *Ph1* even in the monosomic state. The resulting line, designated CS *Ph^1^*, was actively used to induce heterologous translocations in wheat–*Aegilops* hybrids (Aghaee-Sarbarzeh et al., 2000; Kuraparthy et al., 2007b; Chhuneja et al., 2008). Another effective gene-promoter of homoeologous chromosome pairing, the *Hpp-5M^g^*, was identified in the substitution *Ae. geniculata–T. aestivum* line DS 5M^g^#1(5D) and mapped on chromosome 5M^g^S (Koo et al., 2020). A many-fold increase in the frequency of recombinant chromosomes was recorded in a double mutant *ph1b ph1b*/*Hpp-5M^g^ Hpp-5M^g^* (Koo et al., 2017; Koo et al., 2020); the authors also noticed frequent occurrence of translocations with proximal breakpoints or interstitial translocations emerging due to the double recombination event.

## *Aegilops* species as a source of economically valuable traits for improving bread wheat

Disease and pest resistance is among the most important traits transferred into wheat from the related species (Knott and Dvořák, 1976; Valkoun et al., 1985; Marais et al., 2003; Volkova et al., 2008; Olivera et al., 2018; Imadi and Gul, 2019; Sadeghabad et al., 2020; Kumar et al., 2022). According to the latest edition of the Wheat Gene Symbol Catalog (Wheat Gene Catalogue, 2024), over 60 genes conferring resistance to foliar diseases have been introduced from *Aegilops* into bread wheat including 26 genes of leaf rust resistance (caused by *Puccinia triticina* Eriks): *Lr9*, *21*, *22a*, *28*, *32*, *35*, *36*, *37*, *39*, *40*, *41*, *42*, *43*, *50*, *51*, *53*, *54*, 5*6*, *57*, *58*, *59*, *62*, *66*, *76*, *85*, and *LrAc*; 8 of yellow rust resistance (caused by *P. striiformi*s Westend. f. sp. *tritici*): *Yr8*, *17*, *28*, *37*, *38*, *40*, *42*, and *70*; 12 of stem rust resistance (caused by *P. graminis* Pers. f. sp. *tritici*): *Sr32*, *33*, *34*, *38*, *39*, *45*, *46*, *47*, *51*, *53*, *62*, and *66*; and 16 of powdery mildew (caused by *Blumeria graminis* f. sp. *tritici*) resistance genes (*Pm2a*, *2mb*, *12*, *13*, *19*, *29*, *32*, *34*, *35*, *53*, *57*, *58*, *66*, *6Sl*, *Y39*, and *MlUM15*). Aside from them, *Aegilops* also served as a source of resistance to a number of other diseases and pests: cereal aphid (*Schizaphis graminum* Rond.)—*Gb3*, *5*, *7*, and *9*; Hessian fly (*Mayetiola destructor* (Say)—*H13*, *23*, *26*, *27*, *30*, and *34*; cereal cyst nematode (*Heterodera avenae* Woll.)—*Cre2*, *4*, *5*, *6*, *7*, *CreX*, and *CreY*; root rot nematodes (*Meloidogyne* spp.)—*Rkn1*, *2*, and *3*; aphid (*Diuraphis noxia* (Mordvilko)—*Dn3*; *Septoria tritic*i blotch (*Mycosphaerella graminicola* (Fuckel) Schroeter)—*Stb5*; *Septoria nodorum* blotch (*Phaeosphaeria nodorum* (E. Muller) Hedjaroude)—*Sn3*; mosaic virus—*Wsm3*; and others. There is no doubt that the genetic potential of *Aegilops* is far from being exhausted, and new promising sources of economically useful traits for breeding resistant wheat varieties can be found among them (Kishii, 2019; King et al., 2024).

Although *Aegilops* species from the U genome group do not share common genomes with wheat, they have already made a significant contribution to wheat improvement, especially in breeding for pest resistance. One of the most widespread and destructive diseases of bread wheat is rust, which occurs in nearly all regions of wheat cultivation (Singh et al., 2015; Schwessinger, 2017; Lewis et al., 2018; Prasad et al., 2020; Kumar et al., 2022; Patpour et al., 2022). Development of wheat varieties with genetically determined resistance remains the most effective and environmentally sustainable strategy to combat rust (Knott and Dvořák, 1976; Singh et al., 2015; Schwessinger, 2017; Kumar et al., 2022). Notably, several *Aegilops* species from the U genome group have already been donors of resistance genes against leaf, yellow, and stem rust (**Table 2**).

**Table 2:** Genes for economically valuable traits transferred into the wheat genome from the U genome-containing *Aegilops* species.

Donor species/accession	Transferred gene	Location of introgression	Linked markers	Reference
Ae. umbellulata				
TA1851^1^	*Lr9*	T6BS•6BL-6U#1		(Sears, 1956; Wang et al., 2023)
PAU 3732	*Lr76/Yr70*	T5U-5DS	*Xwmc805*	(Chhuneja et al., 2008; Bansal et al., 2017)
Y39	*Pm9*	2U(2B)	*Xgwm257* *Xgwm296* *Xgwm319*	(Zhu et al., 2006)
Ae. biuncialis				
TA7733	*Pm2M^b^*	T2DS.2DL-2M^b^L; Ti2DS.2DL-2M^b^L-2DL	*CL91504* *CL114140*	(Schwessinger, 2017)
Ae. geniculata				
PAU 3537 (TA10437)	*Lr57/Yr40*	T5M^b^-5DS	*Lr57/Yr40-MAS-CAPS16 Xgwm190*	(Kuraparthy et al., 2007a; Kuraparthy et al., 2009; Bansal et al., 2017; Steadham et al., 2021)
TA5599	*Sr53*	T5DL-5M^g^L·5M^g^S	*Xbe442600* *Xbe443201*	(Liu et al., 2011)
TA2899	*Pm29*	?-7DL	*S23M16-246* *S26M26-261*	(Friebe and Heun, 1989; Zeller et al., 2002)
SY159 (PI 487224)	*PmAege7M*	7M^g^(7A)		(Ma et al., 2023)
TA1800	*Hpp-5M^g^*	5M^b^		(Koo et al., 2020)
Ae. kotschyi				
#8078 *T. aestivum–Ae. kotschyi*	*Lr54/Yr37*	2DL		(Marais et al., 2005)
Ae. peregrina				
#680 (from Israel)	*Lr59*	T1AS·1S^P^·6S^P^-6BS	*Xdupw217*	(Marais et al., 2008)
PAU 3519	*LrP/YrP*	5DS	*BS00163889* *5DS44573_snp*	(Narang et al., 2019)
The same	*LrAp*	T6BS.6BL-6U^P^		(Narang et al., 2020)
No. 1 from INRA	*CreX*	T2S^p^-1BL	*Xbarc80* *Xgwm793*	(Coriton et al., 2009)
The same	*CreY*	T3S^p^L-3BL	*Xgwm299* *Xgwm247* *Xgwm181* *Xgwm344*	(Coriton et al., 2009)
The same	*Rkn2*	T3S^p^L-3BL	*OpY16-1065*	(Yu et al., 1992; Yu et al., 1995; Barloy et al., 2000; Coriton et al., 2009)
Ae. neglecta, 4×				
#155	*Lr62/Yr42*	T6AL-6^Aen^L.6^Aen^S		(Marais et al., 2009)
PI 669385	*MlUM15*	7AL	*Xcfa2257* *Xcfa2240*	(Worthington et al., 2014)
Ae. triuncialis				
PAU 3549 (TA10438)	*Lr58*	T2BL-2Ae^t^L	*XksuF11* *XksuH16* *Xbg123*	(Kuraparthy et al., 2007b; Wang et al., 2023)
The same	*LrTr*	T5UL.5US-5AS	*Xgwm156* *Xgwm186*	(Aghaee-Sarbarzeh et al., 2002)
PAU 3462	*LrTri/YrTri*	7BS/7DS	*Xwmc606.*	(Arora et al., 2021)
TR353 derivatives	*Cre7*	n/d		(Romero et al., 1998)
The same	*H30*	T4U^t^-4D	*Acph-U1*	(Martin-Sanchez et al., 2003)
	*Gc1-C1*	2C^t^L		(Endo, 1996)
	*Gc3-C1*	3C^t^		(Endo, 1979)

*Aegilops umbellulata* (**Figure 1D**): The first successful transfer of the leaf rust resistance gene from *Ae. umbellulata* to common wheat was performed by Dr. E.R. Sears (1956). He identified *Ae. umbellulata* accession with high resistance to leaf rust and crossed it with *T. dicoccoides* to produce an amphiploid, which, in turn, was crossed with CS. Following two backcrosses with CS and subsequent selection for resistance, Sears obtained a leaf rust-resistant wheat plant with *Ae. umbellulata* chromosome addition, which carried many disadvantageous phenotype modifications. Based on this line, he obtained an alien isochromosome addition composed of the resistance-determining arms. It was irradiated with X-rays to pollinate normal CS. In the progeny, Sears selected only resistant plants. Over 40 offspring carried one of at least 17 different wheat–*Aegilops* translocations; however, only one cytologically undetectable translocation showed normal transmission through the gametes. The line carrying this translocation was named “Transfer,” while the gene conferring its resistance was designated *Lr9.* C-banding and genomic *in situ* hybridization (GISH) analyses of the “Transfer” showed that the translocation designated T6BS•6BL-6U#1 occurred between homoeologous chromosomes and that the alien fragment is very small and located terminally in the long arm of wheat chromosome 6B (Friebe et al., 1996a). Although this introgression causes some yield reduction (Knott and Dvořák, 1976; Ortelli et al., 1996a; Ortelli et al., 1996b), it was widely adopted in modern wheat breeding. According to GRIS (http://wheatpedigree.net), the *Lr9* is present in 79 common wheat cultivars, including Abe, Arthur-71, Agra, Agripro-Mason, Agripro-Natchez, Chiarano, Clemson 201, Coker-762, Coker-833, Coker-9024, Fundulea-29, McNair-701, Oasis, Pennmore, Riley-67, Sullivan, Tribute, etc., and in 49, it was proven to derive from *Ae*. *umbellulata.* A number of *Lr9-*carrying breeding lines have been developed in the Ural region of Russia and in Kazakhstan (Gultyaeva et al., 2024). Most *Lr9* cultivars were produced and grown in North America, and their ratio is rising with time (Martynov et al., 2015). *Lr9* is still effective in many wheat-growing areas, despite virulent *Pt* isolates emerging soon after the release of the first *Lr9*-carrying cultivars (Huerta-Espino et al., 2008).

Recently, Wang et al. (2023) cloned the *Lr9* gene through mutagenesis and transcriptome sequencing and demonstrated that it encodes an unusual tandem kinase fusion protein. Long-read sequencing of the “Transfer” and *Ae. umbellulata* accession TA1851, which was identified as a putative donor of the *Lr9* gene, enabled reconstruction of the introgressed ~28.4-Mb *Lr9* region and precise localization of translocation breakpoints. The loss of a 5.58-Mb fragment from chromosome 6B, containing 87 high-confidence predicted genes, was identified. This deletion, combined with linkage drag from undesirable *Ae. umbellulata* genes co-introgressed with *Lr9*, may cause yield reduction in translocation-carrying lines (Huerta-Espino et al., 2008). A telomere-to-telomere genome assembly of *Ae. umbellulata* PI 554389 and resequencing 20 other *Ae. umbellulata* genomes identified six new haplotypes of *Lr9* (Singh et al., 2024). Several *T. aestivum*–*Ae. umbellulata* introgressive lines resistant to leaf rust (mediated by the *Lr9* gene) have been recently developed by Tyryshkin and Kolesova (2020).

New genes conferring resistance to leaf and stripe rust were identified in other accessions of *Ae. umbellulata* and used to develop introgressed disease-resistant wheat lines. For instance, Chhuneja et al. (2008) transferred two novel leaf rust resistance genes and a stripe rust resistance gene from *Ae. umbellulata* accession #3732 to bread wheat (**Table 2**). They crossed an amphidiploid WH890 (*Ae. umbellulata* × *T. durum*), line WH890, with CS *Ph^I^* to induce homoeologous pairing between wheat and *Ae. umbellulata* chromosomes. Their F_1_ hybrid was crossed with the susceptible bread wheat cultivar WL711, and resistant plants were selected in the backcross progeny. According to rust reaction and allelism tests, the resistant lines split into two groups. The first group (exhibiting leaf rust resistance at the seedling stage) carried the *Lr9* gene, while the second group harbored at least two novel leaf rust resistance genes (temporarily designated *LrU1* and *LrU2*) and one stripe rust resistance gene (*YrU1*). Targeted GISH analysis revealed that the first lines possessed a complete *Ae. umbellulata* chromosome. Two lines with no apparent linkage drag carried translocations of *Ae. umbellulata* genetic material to the short arm of chromosome 5D. These lines can serve as promising donors of resistance genes for breeding programs (Özgen et al., 2004; Chhuneja et al., 2008).

Genetic mapping revealed that two of the newly identified genes, *Lr76* (*LrUmb*) and *Yr70* (=*YrU1*), are closely linked and located at the distal end of chromosome 5DS, being 7.6 cM distal to the marker *Xgwm190-5D* (Bansal et al., 2017). The introgressed region spanned approximately 9.47 Mb and was predicted to contain six NB-LRR (nucleotide-binding site leucine-rich repeat) (Bansal et al., 2020).

The powdery mildew resistance gene *Pm9* was introduced by Zhu et al. (2006) from *Ae. umbellulata* accession Y39 into bread wheat using a complex combination of crosses. First, they produced a mildew-resistant amphidiploid Am9 by crossing *Ae. umbellulata* × *Triticum carthlicum* and crossed it with the susceptible bread wheat cultivar Laizhou953, followed by three backcrosses with Laizhou953 to obtain a resistant cultivar. Microsatellite analysis revealed a complete 2B(2U) substitution; among others, SSR markers *Xgwm257/185*, *Xgwm296/130*, and *Xgwm319/180* co-segregated with powdery mildew reaction, and *Xgwm296* was “resistance-dominant” (Zhu et al., 2006). All these group 2-specific wheat markers were amplified only in *Ae. umbellulata* Y39 and in the resistant plants, indicating that the resistance was controlled by the *Ae. umbellulata* chromosome, presumably 2U.

*Aegilops biuncialis* possesses a complex of valuable traits that can be used in wheat breeding. It is characterized by high adaptability, in particular salt tolerance, drought tolerance, and heat resistance (**Table 3**). *Aegilops biuncialis* is more drought-resistant than wheat (Molnár et al., 2004). To transfer drought tolerance from *Ae. biuncialis* to bread wheat, wheat cultivar Mv9kr1 was crossed with *Ae. biuncialis* accessions MvGB470 and MvGB1112 (Logojan and Molnár-Láng, 2000). The resulting amphiploids were more tolerant to drought and were able to maintain photosynthetic processes under moderate reduction of water content during osmotic stress (Dulai et al., 2014). The highest drought tolerance was recorded in *T. aestivum*–*Ae. biuncialis* lines Ae9041 and Ae9061 with 6U^b^ chromosome addition (Zhao et al., 2013).

**Table 3:** Agronomically valuable traits of *Aegilops* species containing the U genome potentially useful in wheat breeding.

Agronomically valuable traits	Donor species^*^	Reference
Leaf rust (*Puccinia triticinae*)	Umb, Tri, Kot, Per, Bin, Gen, Col, Neg	(Gill et al., 1985; Valkoun et al., 1985; Manisterski et al., 1988; Antonov and Marais, 1996; Boguslavsky and Golik, 2003; Anikster et al., 2005; Mujeeb-Kazi et al., 2009)
Stem rust (*P. graminis*)	Gen, Per, Neg	(Gill et al., 1985; Valkoun et al., 1985; Manisterski et al., 1988; Boguslavsky and Golik, 2003; Anikster et al., 2005; Mujeeb-Kazi et al., 2009; Olivera et al., 2018)
Stripe rust (*P. striiformis*)	Tri, Kot, Per, Gen, Col, Neg	(Gill et al., 1985; Valkoun et al., 1985; Holubec et al., 1992; Marais et al., 2003; Anikster et al., 2005; Volkova et al., 2008; Chhuneja et al., 2016; Sadeghabad et al., 2020)
Powdery mildew (*Erysiphe graminis*)	Umb, Tri, Kot, Per, Bin, Gen, Col, Neg, Rec, Juv	(Gill et al., 1985; Valkoun et al., 1985; Holubec et al., 1992; Boguslavsky and Golik, 2003)
Root rot, *Fusarium* head blight (*Fusarium* ssp.)	Tri, Gen, Juv	(Boguslavsky and Golik, 2003; Yang et al., 2022)
Yellow spot (*Pyrenophora tritici-repentis*)	Bin, Gen, Col, Neg	(Alam and Gustafson, 1988)
*Septoria nodorum* blotch	Umb, Tri, Bin, Gen, Col, Neg	(Valkoun et al., 1985; Boguslavsky and Golik, 2003)
Hard smut (*Tilletia caries*)	Umb, Tri, Bin, Col, Neg, Rec, Juv	(Boguslavsky and Golik, 2003)
Barley yellow dwarf virus	Tri, Bin, Neg	(Makkouk et al., 1994)
Cereal aphid (*Schizaphis graminum*)	Umb, Per, Col, Neg	(Gill et al., 1985; Holubec and Havliikovi, 1994; Smith et al., 2004)
Russian wheat aphid (*Diuraphis noxia*)	Kot, Bin, Gen	(El Bouhssini et al., 2011)
Hessian fly (*Mayetiola destructor*)	Umb, Tri, Gen, Neg	(Gill et al., 1985; El Bouhssini et al., 1998)
Karnal bunt (*Tilletia indica*)	Tri, Bin, Gen, Col, Juv	(Warham et al., 1986; Harjit-Singh et al., 2000)
Cereal root-knot nematode (*Meloidogyne naasi*) and cereal cyst nematode (*Heterodera avenae* Woll.)	Umb, Tri, Per	(Person-Dedryver et al., 1985; Rivoal et al., 1986)
Drought tolerance	Umb, Bin, Tri, Kot, Per, Gen, Col	(Damania et al., 1992; Benveniste-Levkovitz et al., 1993; Zaharieva et al., 2001; Molnár et al., 2004; Mujeeb-Kazi et al., 2009; Placido et al., 2013)
Heat tolerance	Umb, Tri, Gen, Neg	(Monneveux et al., 2000; Zaharieva et al., 2001; Green et al., 2018)
Frost tolerance	Umb, Tri, Neg	(Shimshi et al., 1982; Monneveux et al., 2000)
Salt tolerance	Umb, Tri, Kot, Bin, Gen, Neg, Juv	(Farooq et al., 1989; Monneveux et al., 2000; Farooq, 2002; Mujeeb-Kazi et al., 2009; Ahmadi et al., 2020; Darkó et al., 2020)
Tolerance to boron toxicity	Bin, Col	(Mujeeb-Kazi et al., 2009; Khan et al., 2021)
High baking quality	Umb, Bin, Gen, Kot, Juv	(Garg et al., 2009; Kozub et al., 2012; Ahmadpoor et al., 2014; Rakszegi et al., 2017; Kumar et al., 2019; Rakszegi et al., 2019)
High grain micronutrient content (Zn^+^ и Mn^+^)	Bin, Kot, Per	(Chhuneja et al., 2006; Rawat et al., 2009; Neelam et al., 2012; Farkas et al., 2014; Kumar et al., 2019)
High grain protein content (HGP)	Bin, Kot, Per	(Chhuneja et al., 2006; Rawat et al., 2009; Farkas et al., 2014; Rakszegi et al., 2017)

Umb, Ae. umbellulata; Tri, Ae. triuncialis; Kot, Ae. kotschyi; Per, Ae. peregrina; Bin, Ae. biuncialis; Gen, Ae. geniculata; Col, Ae. columnaris; Neg, Ae. neglecta; Rec, Ae. recta; Juv, Ae. juvenalis.

*Aegilops biuncialis* is a useful source of genes for salt tolerance. This complex trait occurs via different mechanisms controlled by many genes. According to current concepts, plant tolerance to elevated Na^+^ content can be developed through a limited Na^+^ uptake and transport between roots and shoots, Na^+^ excretion through the cytosol, and plant protection from salt-induced osmotic stress through the accumulation of osmoprotectants (Liang et al., 2018). The latter are water-soluble sugars, proline, and glycine betaine, the main organic substances that accumulate in plant tissues in response to biotic stresses (Ashraf and Foolad, 2007). Testing of parental forms and a series of introgressed *Ae. biuncialis* × *T. aestivum* lines for salt tolerance revealed that the wild type, as well as introgression lines with the addition of chromosomes 2M^b^ and 3M^b^ and translocation of 3M^b^.4BS, showed higher germination potential, shoot and root growth, better CO_2_ assimilation capacity, and lesser chlorophyll degradation upon salt stress compared to the parental wheat form. The paternal *Aegilops* form accumulated less Na^+^ in the roots due to the upregulation of the *SOS1*, *SOS2*, and *HVP1* genes, leading to an increase in the content of proline and a number of sugars (Darkó et al., 2020). Low Na^+^ levels in the leaves were accompanied by an increase in proline content due to enhanced expression of the *NHX2* gene. Plants with chromosome additions 3M^b^ and 3M^b^.4BS were characterized by accelerated accumulation of sugars and proline in the roots, whereas in the chromosome 2M^b^ addition line, an excess of Na^+^ in the leaves was excreted into vacuoles due to overexpression of *HVP1* in the roots and *NHX2* in the leaves. These observations pointed to different mechanisms of salt tolerance controlled by these chromosomes (Darkó et al., 2020). In addition to Na^+^ tolerance, some *Ae. biunicalis* accessions were also tolerant to boron toxicity and, thus, can serve as valuable donors of this trait in wheat breeding (Khan et al., 2021).

*Triticum aestivum* × *Ae. biuncialis* introgressive lines with 3M^b^ and 3M^b^.4BS chromosome additions also showed an increased content of microelements in grain (Farkas et al., 2014), while wheat–*Ae. biuncialis* 1U^b^ addition line carried alleles of high-molecular weight glutenin subunits (HMG-GS), associated with high baking quality (Zhou et al., 2014). The addition of *Ae. biuncialis* group 5 and 7 chromosomes to wheat resulted in an increase in the protein and dietary fiber content in grain, thereby improving the nutritional value of flour (Rakszegi et al., 2017; Rakszegi et al., 2019).

*Aegilops biuncialis* is characterized by high resistance to powdery mildew and moderate resistance to leaf rust, but is affected by Hessian fly and cereal aphid (Gill et al., 1985). Resistance to stem rust in this species turned out to be race-specific (Olivera et al., 2018).

To transfer powdery mildew resistance from *Ae. biuncialis* to bread wheat, Men et al. (2022) crossed disomic 2M^b^-addition CS line TA7733 with CS *ph1b* to induce homoeologous recombination between 2M^b^ and bread wheat chromosomes 2A/2D. This resulted in 65 recombinants, classified into 12 variants using 2M^b^-specific molecular markers. The authors determined the size of the introgressed fragment using GISH and tested recombinant lines for powdery mildew resistance. As a result, a novel resistance gene, temporarily designated *Pm2M^b^*, was identified and localized on the long arm of chromosome 2M^b^ within the interval of fraction length (FL) 0.49 to 0.66 (Men et al., 2022). Molecular markers were developed for this gene to facilitate tracing of this introgression in breeding material, while two recombinants with the smallest introgression size could be of greatest interest for the selection of mildew-resistant wheat cultivars.

Testing of *Ae. biuncialis* for resistance to barley yellow dwarf mosaic virus resulted in the identification of several resistant accessions that originated from Bulgaria; i.e., this species can potentially serve as a donor of resistance genes to this disease (Makkouk et al., 1994).

*Aegilops geniculata* (**Figure 1G**) is one of the most promising donors of agronomically valuable traits including disease and pest resistance (**Table 3**). Currently, at least four *Ae. geniculata-*derived disease resistance genes have been introduced into common wheat.

Leaf rust resistance gene *Lr57* was identified by Aghaee-Sarbarzeh et al. (2002) in the disomic substitution line DS 5M^g^(5D) obtained from a cross between leaf and stripe rust-susceptible wheat cultivar WL711 and the resistant *Ae. geniculata* accession #3537 (=TA10437). To reduce the size of the introgressed fragment, homoeologous recombination between 5D of wheat and *Ae. geniculata* 5M^g^ chromosomes was induced using the *Ph^I^-*mediated system (Aghaee-Sarbarzeh et al., 2002). Putative recombinants were tested for rust resistance and analyzed by GISH and with SSR markers. GISH identified three types of introgressions: one was complete chromosome substitution, and the second was a translocated chromosome composed of the whole short arm and most part of the long arm of 5M^g^ with a small fragment of the wheat chromosome. The third introgression (recombinant line 2K-11-1) was cytologically undetectable (“cryptic” translocation). The recombinant chromosome consisted of the whole short arm and the major part of the long arm of 5D and a small part of 5M^g^L (nearly 15% of the 5M^g^L arm length). The cryptic introgression, which harbors the novel leaf and stripe rust resistance genes *Lr57* and *Yr40*, comprised less than 5% of the 5DS arm (Aghaee-Sarbarzeh et al., 2002; Dhaliwal et al., 2002; Kuraparthy et al., 2007a; Kuraparthy et al., 2009). To map novel genes, the line TA5602 carrying cryptic introgression was crossed with susceptible wheat–*Ae. geniculata* 5M^g^(5D) addition line to develop a mapping panel for identifying target genes. The 5M^g^-specific markers were generated based on data of next-generation sequencing of flow-sorted 5M^g^ chromosome, which allowed localizing *Lr57* and *Yr40* within a physical interval of ~1.5 Mb (Steadham et al., 2021).

Another gene, *Sr53*, conferring resistance to stem rust Ug99 races RKQQC and TTKSK, was transferred from the T5DL-5M^g^L.5M^g^S chromosome to the long arm of bread wheat chromosome 5D by inducing heterologous recombination in a hybrid of translocation line TA5599 × CS *ph1b* (Liu et al., 2011; Kumar et al., 2022). Nine spontaneous recombinants with the reduced introgressed fragment of 5M^g^ were identified. Three recombinants carried interstitial translocations (T5DS.5DL-5M^g^L-5DL), which retained the proximal part (~20%–30%) of the introgressed 5M^g^L segment. Six lines possessed T5DL-5M^g^L.5M^g^S with a shortened 5M^g^L fragment (32%–45% of the total arm length). Based on analysis of the recombinant lines using GISH and 5D- and 5M^g^-specific SSR/STS-PCR markers, all lines were considered as genetically compensated, and three *Sr53*-carrying lines were recommended as new sources of rust resistance (Liu et al., 2011).

Powdery mildew resistance gene *Pm29* was transferred from *Ae. geniculata* to the bread wheat line Pova from the addition line VI *T. aestivum* cv. Poros–*Ae. geniculata* (accession TA2899) (Friebe and Heun, 1989). Using monosomic analysis and AFLP mapping, *Pm29* was localized on the long arm of wheat chromosome 7D (Zeller et al., 2002).

Another novel powdery mildew resistance gene, tentatively designated *PmAege7M*, was identified in the 7M^g^(7A) disomic substitution line W16998, obtained from a cross between CS and mildew-resistant *Ae. geniculata* accession SY159=PI 487224 (Wang et al., 2022; Ma et al., 2023). In addition to resistance, the W16998 exhibited a number of agronomically valuable traits, such as high tillering, high grain number, and 1,000-grain weight, making it a promising breeding material. The line W19513, isolated from the same cross combination and identified as 3M^g^ disomic addition, showed a moderate level of resistance to powdery mildew and high resistance to stripe rust (Wang et al., 2021).

Two CS × *Ae. geniculata* substitution lines (W623 and W637), which were derived from the 7M^g^ disomic addition line W166, showed high resistance to powdery mildew, yellow rust, and *Fusarium* head blight (FHB); resistance to FHB in wheat–*Ae. geniculata* lines was found for the first time (Yang et al., 2022). The alien chromosome was identified as 7M^g^ using GISH, FISH, EST-STS, and PLUG markers for different homoeologous groups. As a result, a disomic substitution 7M^g^(7A) was identified in W623 as well as a disomic substitution 7M^g^(7D) in W637. With the help of functional or linkage markers to FHB resistance genes or QTL, Yang et al. (2022) proved that W623 and W637 possess novel gene(s) controlling resistance to FHB, which are located on chromosome 7M^g^ of *Ae. geniculata*. Both 7M^g^ substitution lines and their parental form W166 were totally immune to powdery mildew race E09 at the seedling and adult stages and nearly immune to stripe rust. Thus, these lines could serve as promising material for developing wheat varieties with improved complex disease resistance.

*Aegilops geniculatа* can also act as a source of tolerance to abiotic stressors. In particular, it can be a potential donor of salt (Gorham, 1990; Farooq, 2002; Colmer et al., 2006) and drought tolerance (Zaharieva et al., 2001; Zaharieva et al., 2003; Pradhan et al., 2012a; Pradhan et al., 2012b), and the *Ae. geniculatа–T. aestivum* 3M^g^ introgressive line was characterized by resistance to copper ions (Landjeva et al., 1998). Chromosomes 1U^g^ and 1M^g^ have a positive effect on the baking quality of wheat flour (Garg et al., 2009; Garg et al., 2016; Rakszegi et al., 2017; Kumar et al., 2019; Rakszegi et al., 2019), while 1U^g^ chromosome addition improved rheological properties of dough by changing the composition and microstructure of gluten (Guo et al., 2021). *Aegilops geniculata* showed high micronutrient content in the leaves and grain (Rawat et al., 2009; Kumar et al., 2019) and can serve as a useful donor of these traits in wheat biofortification.

*Aegilops geniculata* can be used in wheat breeding as a carrier of gametocidal (*Gc*) genes. A new *Gc* gene causing chromosomal breaks in gametophytes, which lost the Gc factor, was identified on chromosome 4M^g^ of *Ae. geniculata* (Kynast et al., 2000). Kwiatek et al. (2017) induced androgenesis at post-meiotic pollen divisions in monosomic plants of hexaploid triticale (AABBRR) with 4M^g^ addition, followed by production of dihaploids (DHs), to maintain the chromosome aberrations caused by the gametocidal action. As a result, they obtained 41 DH lines, 17 of which carried different types of chromosomal aberrations in a homozygous state. *Aegilops geniculata* also possess genes affecting chromosome pairing. Thus, Koo et al. (2017) identified a new homoeologous pairing promoter gene(s) *Hpp-5M^g^* on the chromosome 5M^g^ in the CS DS5M^g^#2(5D) double disomic substitution line. The *Hpp-5M^g^* gene significantly increased chromosome pairing and recombination frequency between wheat and homoeologous alien chromosomes, especially when combined with *ph1b*, permitting to induce of crossing over not only in the distal but also in the proximal regions of chromosomes, resulting in proximal and interstitial translocations (Koo et al., 2020).

*Aegilops kotschyi* possess a number of valuable traits that are in demand in breeding (**Table 3**). Antonov and Marais (1996) discovered *Ae. kotschyi* accession from Israel resistant to brown and yellow rust pathotypes common in South Africa (Antonov and Marais, 1996; Marais et al., 2003). They crossed it with CS and tested their offspring for rust resistance. The resistant form was backcrossed by CS, and the line 8078 containing a pair of *Ae. kotschyi* addition chromosomes was selected. The line had tenacious glumes inherited from the wild parent. In wheat, this trait is controlled by the dominant *Tg* gene localized on chromosome 2DS introgressed from *Ae. tauschii* (Wheat Gene Catalogue, 2024); thus, the alien chromosome could belong to homoeologous group 2. The line was crossed with three monosomic lines (CS M2A, CS N2B, CS M2D), and the resistant F_1_ plants with 2*n*=42, probably with group 2 wheat/group 2 *Aegilops* monosomic chromosome substitution, were selected from each cross and backcrossed as male parent to CS or another susceptible variety. The TF_1_ progeny was tested and the resistant plants were raised to TF_2_ families, which were again tested for leaf rust resistance. The alien chromosomes in monosomic addition lines (2*n*=42w+1Ae) always showed a low transmission rate, which was confirmed by screening TF_1_ and TF_2_ progenies for rust resistance.

Based on this knowledge, Marais et al. (2005) proposed that a higher transmission rate could be an indicator of compensating translocation with alien chromosome carrying resistance genes. Indeed, one population, S14, obtained in a test cross of the double monosomic 2D/*Ae. kotschyi* group2//CS contained 96% of the resistant plants and apparently carried a translocated chromosome (Marais et al., 2005). The authors selected resistant plants with 2*n*=42, which displayed normal threshability, shorter stature, and a much shorter growth period compared to the addition line. These plants presumably carried Robertsonian translocation between T2DS and the long arm of an undefined group 2 chromosome of *Ae. kotschyi* (Marais et al., 2005; Heyns et al., 2011). The translocated chromosome showed normal transmission through female gametes, but strong preferential transfer of male gametes carrying the resistance genes (Marais et al., 2005). The authors suggested that *Ae. kotschyi* chromosome present in the addition line 8078 possessed two linked genes conferring resistance to leaf rust and yellow rust designated *Lr54/Yr37.* These genes were localized on the long arm of *Ae. kotschyi* chromosome, which was translocated on 2DS in line S14 (Kuraparthy et al., 2007a). To reduce the size of the introgressed chromatin, Heyns et al. (2011) induced recombination between T2DS-2Ae^k^L and 2DL by using the CS *ph1b* system and obtained 10 stable recombinant lines with shortened alien fragment. Recombinant translocations presumably occurred due to single crossovers, leading to the replacement of a distal part of the alien chromosome fragment with wheat chromatin. Translocation recombinants were characterized using microsatellite, SCAR, and AFLP markers for 2DL. The resistant recombinants were classified into three categories according to the length of alien chromatin retained in the recombinant chromosomes, and the shortest alien fragment occupying the proximal half of the chromosome was detected in recombinant #74 (Heyns et al., 2011). To assess the perspectives of *Lr54/Yr57* in wheat breeding, the authors developed near-isogenic lines of rec. #74 in adapted varieties and developed a dominant SCAR marker for target genes and several microsatellite markers linked to *Lr54/Yr57* to trace them in breeding material (Heyns et al., 2011).

A similar translocation, but between the rye chromosome 2RS and the 2S^k^L chromosome of *Ae. kotschyi*, has later been produced by Ulaszewski et al. (2019) by crossing the ditelosomic (40T + D2RS + D2RL) line of triticale cv. Sekundo × monosomic substitution line of triticale carrying a single copy of 2S^k^ chromosome. GISH confirmed the presence of Robertsonian translocation between rye and *Aegilops* chromosomes, whereas FISH identified *Ae. kotschyi* chromosome as 2S^k^ (Ulaszewski et al., 2019). The application of molecular markers developed earlier for *Lr54/Yr57* genes (Heyns et al., 2011) to recombinant triticale lines revealed an amplicon of the expected size, which indicated that generated 2S^k^.2R RobTs translocation carried the same resistance genes and the recombinant chromosome described by Marais et al. (2005) composed of 2DS and *Ae. kotschyi* chromosome 2S^k^L.

*Aegilops kotschyi* is characterized by high grain microelement content (HGMC) compared to cultivated wheat and can be used as a valuable donor in wheat breeding (Chhuneja et al., 2006; Rawat et al., 2011; Verma et al., 2016a; Verma et al., 2016b; Kumar et al., 2019). To transfer GMC, Rawat et al. (2009) crossed the CS *Ph^I^* line and wheat cultivars lacking the *Ph^I^* gene with *Ae. kotschyi*, which was chosen from 90 *Triticum* and *Aegilops* accessions based on the three- to fourfold higher content of iron and zinc and ~33% higher protein content in grains. Most F_1_ hybrids produced in these crosses were sterile because of meiotic disturbances (Rawat et al., 2009). The sterile F_1_ hybrids were backcrossed as female parent with wheat, and some of the obtained BC_1_ hybrids and their BC_2_ progeny showed nearly 60% higher grain ash iron and zinc content than the recurrent wheat parent, suggesting that the useful variability of *Ae. kotschyi* for higher ash Fe^+^ and Zn^+^ concentration has been transferred to wheat.

Thirteen BC_2_F_2_ and BC_1_F_3_ lines obtained from the cross CS (*Ph^I^*) × *Ae. kotschyi* (acc. 396), which were morphologically similar to the recurrent wheat parent and showed high microelement and protein content in grain, were selected for the assessment of introgressions by using morphological traits, HMW-GS profiles, wheat microsatellite markers, meiotic analysis, and GISH (Tiwari et al., 2010; Rawat et al., 2011). The authors found that HGMC correlates with the presence of addition and substitution of group 2 and 7 chromosomes of *Ae. kotschyi*. The 2S^k^ addition was found in 10 out of 13 derivatives with the enhanced grain protein and Zn^+^ concentration, and the group 7 chromosome introgression was identified in 10 lines (Rawat et al., 2011). Molecular markers for group 2 and 7 chromosomes revealed that the introgressions (additions and substitutions) were represented by whole chromosomes. Besides 2S^k^ and 7U^k^/7S^k^, hybrid lines may contain other *Ae. kotschyi* chromosomes, but they have no effect on the target traits. The plants with exceptionally high grain iron and zinc contents possessed additional HMW-glutenin subunits inherited from *Ae. kotschyi* #396, showing that 1U^k^/1S^k^ chromosome(s) introgressions conferred very high grain iron and zinc content in the derivatives. These chromosomes also had the greatest effect on increasing grain micronutrient content.

One line containing the whole chromosome 7S^k^ and a translocated 2S^k^ chromosome with a fragment of wheat chromosome on the tip of the short arm did not show any increase in grain protein content (GPC); it had waxy leaves and spikes. The low protein content in this derivative suggests that the regulatory gene for high GPC in *Ae. kotschyi #*396 may be on the telomeric end of chromosome 2S^k^S (Rawat et al., 2011).

To transfer the HGMC from *Ae. kotschyi* to wheat, Verma et al. (2016b) employed the system of induced homoeologous chromosome pairing by crossing *Ae. kotschyi* (acc. 3790) with Pavon-mono-5B variety. They obtained F_1_ plants with 35 and 34 chromosomes (monosomic for 5B), which were completely sterile. The F_1_ plants with 34 chromosomes (lacking 5B) showed high homoeologous pairing. They were crossed with the wheat variety PBW343 (*Lt24 + Yr36*), and the resulting partially fertile BC_1_F_1_ plants were backcrossed by PBW343 and their BC_2_F_1_ progenies were screened for pollen viability, chromosome number, and meiotic chromosome pairing. The somatic chromosome number in the offspring varied from 43 to 60, and different lines possessed introgressions such as whole U^k^/S^k^ chromosome(s) as well as one to several terminal or interstitial translocations of variable size involving both wheat and *Aegilops* chromosomes. Analysis of HMW-GS profiles of the BC_2_F_1_ plants allowed the identification of three lines with 1U^k^/1S^k^ introgression, all showing higher grain mineral micronutrient content. Some BC_2_F_2_ plants exhibited a 125% increase in Fe^+^ content (MB-27, 2*n*=42, 19^II^ + 4^I^) and a 158% increase in Zn^+^ content (BM-35, 2*n*=57, 23^II^ + 11^I^) relative to the PBW343 variety and can be used for wheat improvement.

*Aegilops kotschyi* can also be used as a source of resistance to unfavorable abiotic factors: high temperatures, salinity and drought (Benveniste-Levkovitz et al., 1993; Bocianowski and Prażak, 2022), and water stress (Shimshi et al., 1982). The cytoplasm of some *Ae. kotschyi* accessions is capable of inducing cytoplasmic male sterility (thermo-sensitive male sterility based on cytoplasm, K-TCMS), which is of considerable interest for hybrid wheat breeding (Mukai and Tsunewaki, 1979; Gupta et al., 2019; Li et al., 2019; Fan et al., 2024). Alloplasmic lines of bread and durum wheat with *Ae. kotschyi* cytoplasm can also be used as haploproducers (Tsunewaki et al., 1974; Mukai and Tsunewaki, 1979; Fan et al., 2024).

*Aegilops peregrina* (**Figure 1F**) is characterized by high resistance to a number of biotic and abiotic stressors. To date, genes controlling resistance to leaf rust (*Lr59*, *LrP*, and *LrAp*), stripe rust (*YrP*) (Marais et al., 2008; Marais et al., 2010a; Pirseyedi et al., 2015; Narang et al., 2018; Narang et al., 2019; Narang et al., 2020) and still uncharacterized dominant gene of stripe rust resistance (Liu et al., 2010), cereal cyst nematode (*CreX* and *CreY*) (Coriton et al., 2009), root-knot nematode (*Rkn2*) (Yu et al., 1990; Yu et al., 1995), and uncharacterized powdery mildew resistance gene (Spetsov et al., 1997) have been transferred from this species to the wheat genome.

The leaf rust resistance gene *Lr59* was introgressed from *Ae. peregrina* to wheat chromosome 1AL. Resistant *Ae. peregrina* accession 680 from Israel was used to pollinate CS followed by chromosome doubling in hybrids by colchicine treatment. The resulting C_1_ plants were backcrossed by CS and by the wheat breeding line W84-17; each generation was tested for leaf rust resistance against five South African pathotypes (Marais et al., 2008). The *Ae. peregrina*-derived resistance was found to be controlled by monosomic addition chromosome (in CS background) and by a translocation chromosome (in W84–17 background). The translocation occurred spontaneously in the line selected from the 4th backcross with W84-17. The addition chromosome was assigned to homoeologous group 1, which agreed with the cytogenetic analysis—the added chromosome showed C-banding pattern similar to the 1S^p^ of *Ae. peregrina* (Friebe et al., 1996b). The translocation chromosome had normal female transmission, while male transmission favored resistant gametes. Meiotic, monosomic, and microsatellite analyses showed that the fragment of *Lr59*-carrying *Ae. peregrina* chromosome constituted almost the entire long arm of 1A; i.e., this translocation is most likely Robertsonian (Marais et al., 2008). Later, GISH revealed that the terminal region of the long arm of the full-length *Lr59* translocation belonged to wheat chromosome; i.e., this was an interstitial translocation. Further study confirmed that the distal part of the original translocation is homoeologous to subtelomeric region of 6BS, so the structure of the rearranged chromosome can be described as 1AS-1L^P^-6S^P^-6BS (Pirseyedi et al., 2015).

To reduce the size of the alien segment, plants heterozygous for the full-length *Lr59* translocation and homozygous for CS *ph1b* mutation were pollinated with either nulli-1A/tetra-1B or nulli-1A/tetra-1D CS plants. Eight potentially useful recombinant resistant lines have been found among selfed F_2_ and F_3_ progenies (Marais et al., 2010a). The obtained recombinants and resistant test-cross F_1_ progeny monosomic for 1A were analyzed using microsatellite markers to localize *Lr59* on genetic and physical maps. Eight of the resulting recombinants, which inherited rust resistance, differed in size and localization of the introgressed region. Two resistant plants were nullisomic for 1A; thus, the *Lr59* fragment was retained on a different chromosome. Probably, this gene was transferred to other wheat chromosome due to allosyndetic homoeologous recombination (Marais et al., 2010a). To identify the non-1AL chromosome(s) involved in the allosyndetic recombination events, the authors built a genetic map of one of the seven recombinants, which did not involve 1AL. The *Lr59* was assigned to the linkage group 6B; the respective locus was mapped 0.5 cM distally from co-segregating SNPs *IWA1495*, *IWA6704*, *IWA2098*, and *IWA969.* Eleven additional SNPs were mapped within an interval 0.5–6.5 cM proximally of *Lr59* (Pirseyedi et al., 2015). The authors showed that a homoeoallele of *Xdupw217* was associated with the *Lr59* alien insert and therefore can be used to trace the resistance gene in breeding populations. By using GISH, the authors found that all—the original and seven resistant recombined *Lr59* translocations—contained a small, similar-sized subterminal wheat chromosome segment and recommended recombinant line Lr59–151 with the smallest introgressed segment for further use in wheat breeding.

Two novel linked genes conferring leaf and stripe rust resistance temporarily designated *LrP* and *YrP* have been introgressed to common wheat from another *Ae. peregrina* accession (pau3519). Narang et al. (2018) crossed it with CS *Ph^I^* to induce homoeologous recombination and backcrossed the F_1_ hybrid by the rust-susceptible wheat cultivar WL711. Their hybrids were tested for rust resistance, and the resistant plants were backcrossed again by WL711. The resistant BC_1_F_1_ plants with good agronomic performance have been selfed over five generations to develop stable homozygous BC_2_F_6_ introgression lines (IL). Two lines, IL pau16058 and IL pau16061, with chromosome number 2*n*=42 were selected for further analyses. They were screened for resistance against six *Puccinia triticina* and two *P. striiformi*s f. sp. *tritici* pathotypes at the juvenile stage and against a mixture of pathotypes of both pathogens prevailing in India at the adult plant stage. IL pau16061 was resistant only to the leaf rust, while IL pau16058 showed resistance to both diseases at all stages of development (Narang et al., 2018). Microsatellite analysis identified a number of alien introgressions of different size in both lines: in IL pau16061, the largest introgression was detected on homoeologous group 6, followed by group 2, whereas in IL pau16058, the largest introgression was observed on homoeologous group 2, followed by group 5. According to SSR analysis, the alien fragment, controlling resistance in IL16058, was translocated to group 5 chromosome of wheat. Later, based on nulli-tetrasomic analysis, the location of the resistance genes was specified to chromosome arm 5DS (Narang et al., 2019). Introgression showed no deleterious phenotypic effects; thus, Narang et al. (2018) suggested that it involved a small alien segment with minimum linkage drag and might be compensatory.

The IL pau16058 was then crossed with the susceptible wheat variety WL711 to develop F_2:3_ mapping population (Narang et al., 2019). Analysis of this population revealed that leaf and stripe rust resistance were inherited as dominant co-segregating traits and therefore were controlled by two independent genes, temporarily designated *LrP* and *YrP*. Genomic origin of the introgressed *Ae. peregrina* fragment was defined using COS and KASP markers, designed for the U genome chromosomes. The results showed that the introgression carrying leaf rust and stripe rust resistance genes in IL pau16058 probably originated from the short arm of 5U^p^ chromosome of *Ae. peregrina* (Narang et al., 2019).

Using the Illumina Infinium iSelect 90K wheat array and resistance gene enrichment sequencing (RenSeq) markers, the *LrP* and *YrP* genes were mapped within the 4.19-cM stretch in the distal part of 5DS containing eight SNPs and one microsatellite marker. *LrP* and *YrP* co-segregated with markers *BS00163889* and *5DS44573_snp* and were flanked by SNP markers *BS00129707* and proximally *5DS149010* (Narang et al., 2019).

Another dominant gene conditioning leaf rust resistance at all stages of plant development, temporarily designated *LrAp* (Narang et al., 2020), was found in the second line, IL pau16061, isolated earlier by Narang et al (Narang et al., 2018). Chromosome localization of the introgressed fragments was determined using GISH with genomic DNA of *Ae. umbellulata* (U^p^) and *Ae.* sp*eltoides* (S) as probes (Narang et al., 2020). Two introgressions have been identified: one from the S^p^ and the other from the U^p^ chromosomes. Rehybridization of the same metaphase cells with chromosome-specific probes showed that the U^p^ genome segment was introgressed to the end of the long arm of wheat chromosome 6B and the translocation can be described as 6U^p^L-6BL. This was a compensating translocation and the novel leaf rust resistance gene temporarily designated *LrAp* was localized on the genetic map of 6BL using KASP markers. Alien segment spans along the 2.91-Mb region at the telomeric end of wheat chromosome 6BL (Narang et al., 2020). The S genome introgression was observed in both resistant and susceptible plants, and therefore, it was not associated with resistance.

Spetsov et al. (1997) transferred resistance to powdery mildew from unspecified *Ae. peregrina* accession of the IWS-General Toshevo collection to the wheat cultivar Rusalka. According to cytological and electrophoretic analyses of HMW glutenins, resistance was controlled by chromosome 1U^р^, introgressed either as a 1U^p^ chromosome addition or 1U^p^(1B) substitution (Spetsov et al., 1997).

Besides the resistance to foliar disease, some *Ae. peregrina* accessions showed resistance to cereal cyst nematode (CCN) caused by *Heterodera avenae* and root-knot nematode (RKN) caused by *Meloidogyne naasi* (Person-Dedryver et al., 1985; Yu et al., 1990; Yu and Jahier, 1992; Yu et al., 1992; Yu et al., 1995; Barloy et al., 2000; Coriton et al., 2009). Person-Dedryver at al (Person-Dedryver et al., 1985). evaluated the collection of wild wheats for nematode resistance and identified several resistant accessions of *Ae. umbellulata* and *Ae. peregrina.* Resistance of *Ae. peregrina* was due to the ability to suppress the development of larvae into females and nematode reproduction (Person-Dedryver et al., 1985; Yu et al., 1990). CS was pollinated with resistant *Ae. peregrina* accession No. 1, and the F_1_ hybrid was backcrossed twice by wheat cultivars Lutin and Rescler. The BC_2_ plants were then selfed up to the BC_2_F_5_ generation, and at each stage, all plants were evaluated for nematode resistance and chromosome number. Two disomic addition lines have been isolated in the progeny from this cross: the BC_2_F_2_ line X35 was resistant to both nematodes (*CreY* and *Rkn2*, previously designated *Rkn-mn1*), and line N was resistant to CCN (*CreX* gene) (Jahier et al., 1998; Coriton et al., 2009). The addition line LX was obtained via three backcrosses of the resistant addition line X35 with the susceptible wheat cultivar Lutin. The stable resistant translocation line X8 was also derived from X35; all X8 plants had 42 chromosomes and showed regular chromosome pairing in meiosis. Yu et al. (1990) suggested that the resistance to root-knot nematode in X8 and X35 is controlled by a single dominant gene *Rkn2* (previously *Rkn-mn1*). As a similar resistance gene was earlier revealed in some accessions of *Ae. longissima*, the donor of *Ae. peregrina* S^p^ genome, the authors proposed that *Rkn-2* could be located on the S^p^ genome chromosome. Monosomic analysis and telocentric mapping of the translocation line X8 and the addition line LX showed that the *Ae. peregrina* segment carrying the *Rkn2* and *CreY* genes was transferred on wheat chromosome 3B (Yu et al., 1995; Barloy et al., 2000). The alien segment was located distally on 3BL, and *Rkn2* and *CreY* were independent of the centromere (Yu et al., 1995). GISH and SSR mapping (Coriton et al., 2009) were fully consistent with this suggestion. In wheat–*Ae. peregrina* addition line LX, four 3BL-specific SSR markers and three SSRs of 7BL, 2BS, and 4BS were detected. In the translocation line X8, all SSRs developed for 3BL amplified products specific for *Ae. peregrina*, while all wheat homoeoloci were absent. Based on these data, Coriton et al. (2009) proposed that a recombination event between the additional chromosome of LX line and wheat took place within the distal part of 3BL.

Jahier et al. (1998) suggested that a segment of *Ae. peregrina* chromosome carrying the CCN resistance gene (*CreX*) in a second line—D, a derivative of line N, was introgressed into unknown wheat chromosome. FISH identified the additional *Ae. peregrina CreX*-chromosome as 6S^p^; however, SSR markers amplified on this chromosome were specific for groups 1, 2, 4, and 6 (Coriton et al., 2009). The SSRs of group 2 were assigned to deletion bins of distal, terminal, and centromeric regions of 2AS or 2DS; thus, the authors proposed that the addition chromosome should be classified as 2S^p^. A *CreX-*homozygous line D3 with a much shorter *Ae. peregrina* segment was obtained by backcrossing translocation line D by Lutin (Barloy et al., 2000). Based on microsatellite analysis, the *CreX* gene in D3 line was translocated on 1BL being in the vicinity to the *Xgwm818* SSR marker (Coriton et al., 2009).

In addition to resistance genes, *Ae. peregrinа* has a number of valuable traits that may be utilized in breeding (**Table 3**). According to Damania et al (Damania et al., 1992), *Ae. peregrinа* is one of the most drought-tolerant species in the genus *Aegilops.* Liu et al. (2015) tested over 80 wheat–*Aegilops* introgressive lines developed on a CS background and found that wheat–*Ae. peregrina* 4S^p^ and 3U^p^ addition line showed excellent performance under drought stress and should therefore be deeply examined and included in breeding programs for drought resistance. According to Farooq et al (Farooq et al., 1989), *Ae. peregrinа* also had a high potential for enhancing the salt tolerance of wheat. Introgression of group 4 and 7 chromosomes doubled the grain microelement content in the respective addition lines compared to the recipient variety (Neelam et al., 2012). In addition, the ability of *Ae. peregrinа* cytoplasm to induce cytoplasmic male sterility (Mukai and Tsunewaki, 1979) can be utilized in hybrid wheat breeding.

*Aegilops neglecta*: Unlike most species from the U genome group, *Ae. neglecta* and *Ae. recta* have been little used for wheat improvement. Only three successful transfers of the resistance genes, particularly leaf and stripe rust resistance *Lr62/Yr42* (Marais et al., 2009), other leaf and stripe rust resistance genes not characterized yet (Bai et al., 1994), and powdery mildew resistance gene *MlUM15* (Marais et al., 2005), from these species to bread wheat have been reported.

A dominant powdery mildew resistance gene, temporarily designated *MlUM15*, was introgressed from tetraploid *Ae. neglecta* accession TTCC 223 (collected by D. Marshall, USDA-ARS in 1992 on a rocky hillside near the town of Karamusa in north-central Turkey) to common wheat (Marais et al., 2005). The winter bread wheat cultivar “Saluda” was crossed with *Ae. neglecta* followed by two backcrosses by the recurrent parent and subsequent selfing. The derived BC_2_F_8_ line NC-UM15 was resistant to powdery mildew, which is supposed to be controlled by a novel resistance gene. To map this gene, Worthington et al. (Worthington et al., 2014) developed a mapping population by crossing NC-UM15 × Saluda with subsequent selfing of the F_1_ plants to produce F_2_ seeds, whose 198 F_2:3_ families were tested for disease resistance. DNA was isolated from all F_2_ plants, which were used for developing F_2:3_ families. Depending on the testing results, the F_2:3_ families were classified as homozygous resistant, homozygous susceptible, or segregating. Worthington et al. (Worthington et al., 2014) pooled DNA isolated from 1) 10 F_2:3_ families with consistently resistant phenotypes, 2) 10 families with consistently susceptible phenotypes, 3) resistant parents, and 4) susceptible parents. These groups were screened using more than 300 SSR markers covering the whole genome of wheat. Markers showing polymorphism between the resistant parent + bulk, on one hand, and the susceptible parent + bulk, on the other, were preliminarily considered as linked to the resistance gene and were taken to screen the entire F_2_ population. Three dominant SNP markers, *IWA2929*, *IWA4434*, and *IWA8057*, were linked in coupling, and *Xcfa2257* was linked in repulsion with the resistance allele. Eleven SSR, STS, and SNP markers provided a basic map for calculating recombination frequencies, and the resistance gene *MlUM15* was mapped to a 1.2-cM interval between *Xcfa2257* and *Xcfa2240.* The physical location on the tip of 7AL was confirmed by analysis of CS aneuploid lines (Marais et al., 2005).

Two linked genes conferring resistance to leaf and yellow rust have been introgressed to common wheat from *Ae. neglecta* #155 (Marais et al., 2009), which was selected among 877 *Aegilops* accessions belonging to 27 species as highly resistant to leaf rust (Antonov and Marais, 1996). This accession was crossed with CS, and their resistant F_1_ hybrid was backcrossed to CS four times to develop a nearly isogenic line with a shortened culm (CS-S). Following five backcrosses by wheat, a monosomic addition plant (8048) was obtained, and its progeny was selfed and tested for disease resistance (Marais et al., 2009). A broad range of infection types has been observed, probably because the gene designated *Lr62* was localized on the addition chromosome assigned to group 3 based on RFLP analysis (Marais et al., 2003). The disomic addition line, 8048-44, was selected from 8048. The *Ae. neglecta* addition chromosome showed mixed homoeology to wheat chromosomes. Its short arm and a proximal part of the long arm were homoeologous to wheat group 6, but a distal region on the long arm was derived from group 3 *Ae. neglecta* chromosome (Marais et al., 2010b). The line 8048 had an extremely poor agrotype and expressed strong hybrid necrosis, but was highly resistant to leaf rust.

On the next step, Marais et al. (2009) aimed to transfer the resistance gene(s) to the homoeologous wheat chromosome and characterize the introgression using genetic markers. Heterologous chromosome pairing was induced by crossing a monosomic addition plant (derived from a backcross of 8048 × CS-S) with a CS double monosomic 3B/5B plant followed by selection of the resistant double monosomic F_1_ progeny with 19″ + 3′. Hybrids were crossed with CS *ph1b* and with the susceptible tester line. The obtained progeny was scored for disease resistance, the resistant plants were selfed, and their F_2_ progeny was scored again for resistance, fertility, and the absence of hybrid necrosis (Marais et al., 2009). Segregation ratio (3:1) obtained in the F_3_ progeny implied that the resistance was controlled by translocation rather than by the addition chromosome. Test-crosses with three CS lines monosomic for 3A, 3B, 3D revealed that the translocation involved wheat chromosome from a different genetic group. The respective wheat chromosome was first searched using monosomic and nulli-tetrasomic analysis, and after that, wheat chromosomes, selected as possible candidates, were tested with arm-specific microsatellite markers. Based on this analysis, wheat chromosome was identified as 6A (Marais et al., 2009). The spontaneous exchange between the addition chromosome in 8048–44 and 6AL of wheat that gave rise to the full-length *Lr62* translocation probably occurred within group 6 homoeologous regions, close to and proximally from the chromosome 3 translocation breakpoint (Somo et al., 2017). As a consequence of translocation, all group 3-derived chromatin on *Ae. neglecta* addition chromosome was replaced with wheat 6AL chromatin.

The addition line 8048–44 also carried a promising yellow rust resistance gene (*Yr42*) effective against South African pathotypes of *P. striiformis* at the seedling stage (Marais et al., 2009). Resistance was not co-transferred in the translocation event and thus may occur within the group 3 homoeologous region. Therefore, it was possible to transfer this gene by inducing homoeologous chromosome pairing.

The *Lr62* gene was efficient against a broad range of South African and Canadian pathotypes of *P. triticina*; however, its carrier had poor agronomical performance. To increase the commercial attractiveness of the obtained translocations, Marais et al. (2010b) attempted to reduce their size by inducing heterologous chromosome pairing. Plants heterozygous for *Lr62/Yr42* translocation and lacking the *Ph1* locus were crossed with CS nulli-6A/tetra-6B or nulli-6A/tetra-6D plants. Resistant (*Lr62*) test-cross F_1_ progeny was evaluated for the presence of three specific SSR markers, resulting in the identification of 41 recombinants. The *Lr62/Yr42* was localized toward the distal end of 6AS, which was homoeologous to the translocated chromosome. Major structural differences found between the CS *ph1b* mutant and the translocated chromosome 6A were caused by a duplication that was probably inherent in CS *ph1b*. Four promising recombinants, which retained both *Lr62* and *Yr42* within a comparatively small introgressed region at the 6AS terminus, were isolated. Subsequent analysis of the recombinants and the original *Lr62* line using 6A-specific microsatellite markers and GISH showed that in three lines, the exchanges occurred near the telomeric region of 6AS (Somo et al., 2017). As recombinant lines retained the *Gli-A2* locus, their translocations should not adversely affect baking quality and can be recommended for practical use (Somo et al., 2017).

There was only one report on the introgression of agronomically valuable traits from hexaploid *Ae. recta* to wheat. Thus, Bai et al. (1994) crossed six *Ae. recta* accessions with good resistance to leaf and stem rust with two susceptible durum and four susceptible bread wheat cultivars. All F_1_ hybrids expressed *Ae. recta-*derived resistance to both diseases. The F_1_ hybrids were backcrossed to their wheat parents to produce BC_1_F_1_ plants, which had a much lower seed set (0%–7.14%) compared to F_1_ (seed set 12.50%–78.33%); both characters varied depending on genotypes of parental species. Meiotic analysis showed that the low female fertility of the F_1_ hybrids was caused by poor chromosome pairing, and only gametes with complete or nearly complete genomes were viable. The BC_1_ plants, obtained only from backcrosses of *Ae. recta* to common wheat, were backcrossed to the susceptible wheat parent one or more times until the fertility was recovered. The selfed progeny was tested for disease resistance, and three families containing resistant plants have been selected.

Nearly half of the BC_1_F_1_ plants (17 vs. 39) had chromosome numbers close to the expected (2*n*=53–56), whereas chromosome numbers in the remaining 22 lines ranged from 44 to 52. The BC_2_F_2_–BC_2_F_4_ plants were segregated for disease resistance. Most susceptible lines had 2*n*=42 (ranged from 40 to 44) and formed 21 bivalents in meiosis. Chromosome number of the resistant lines varied depending on cross combination from 2*n*=43 or 44 with 21″ + 1′ or 21″ + 1″ in one combination to 43–50 in two others (Bai et al., 1994). These data indicated that the resistance genes must be located in alien chromosomes added to wheat. *Aegilops recta* chromosome associated with disease resistance was identified using 14 RFLP markers specific for seven homoeologous groups of wheat (Bai et al., 1994). The addition chromosomes carrying leaf resistance genes in lines 1 and 2 were assigned to homoeologous groups 5 and 2, and the stem rust resistance gene was localized on the group 7 chromosome of *Ae. recta* (Bai et al., 1994).

Genetic potential of *Ae. neglecta*, however, is not fully exploited (**Table 3**). More than 80% of *Ae. neglecta* accessions were resistant to Ug99 races TTKSK, TTTTF, and TRTTF (Olivera et al., 2018). Some accessions showed multiple aphid resistance (El Bouhssini et al., 2011) and can be useful donors of this trait.

*Aegilops triuncialis* is geographically one of the most widespread *Aegilops* species, which is adapted to a broad range of biotic and abiotic stressors (**Table 3**). Many accessions of *Ae. triuncialis* showed high resistance to diseases (Gill et al., 1985; Harjit-Singh et al., 2000) and pests (Romero et al., 1998) and served as donors of these traits in wheat breeding (**Table 2**). Thus, Harjit-Singh et al. (2000) crossed susceptible wheat cultivars WL711 with *Ae. triuncialis* acc. 3549 (=TA10438) with complex resistance to leaf rust, Karnal bunt, powdery mildew, and cereal cyst nematode (CCN) and backcrossed the sterile F_1_ hybrid by the recurrent wheat parent. The resistant BC_2_/BC_3_ plants gave rise to two groups of derivatives. One group with 2*n*=42 was resistant to CCN and powdery mildew and moderately resistant to leaf rust, while the second group with 2*n*=44 possessed leaf rust, Karnal bunt, and powdery mildew resistance. C-banding analysis of the first group identified a complete 5A(5U^t^) chromosome substitution, which was confirmed by GISH. GISH also revealed an additional, segregating wheat–*Ae. triuncialis* recombinant chromosome, possessing a small terminal translocation from the U^t^ chromosome to the short arm of wheat chromosome (Aghaee-Sarbarzeh et al., 2002). GISH on the second group of introgressive lines (2*n*=44) detected disomic addition of acrocentric *Ae. triuncialis* chromosome and Robertsonian translocation between the unknown wheat (long arm) and *Ae. triuncialis* (short arm) chromosomes (Harjit-Singh et al., 2000). The authors suggested that gene(s) conferring resistance to leaf rust, Karnal bunt, and powdery mildew could be linked and located either on wheat–alien translocated chromosome or on the alien acrocentric chromosome. Alternatively, gene(s) for resistance to one or two diseases could be split between different introgressions (Harjit-Singh et al., 2000).

Kuraparthy et al. (2007b) transferred the leaf rust resistance gene, designated *Lr58*, from *Ae. triuncialis*, #3549 to wheat. The introgression line was developed by crossing the susceptible cultivar WL711 with the resistant *Ae. triuncialis* accession #3549 and backcrossing the resistant F_1_ plants by WL711 (Harjit-Singh et al., 2000; Aghaee-Sarbarzeh et al., 2002). Resistant BC_1_F_1_ plants were selected, backcrossed by WL711, and selfed to develop BC_3_F_11_ lines (starting from BC_2_F_1_ and BC_3_F_1_, only resistant plants with a complete set of wheat chromosome were selected for subsequent selfing). The resistant BC_3_F_11_ line TA5605 had normal plant growth and development. This line, its parental forms *Ae. triuncialis* #3549, WL711, and CS, and wheat cultivar Jagger were subjected to molecular-genetic and cytogenetic analyses. The segregation ratio 3:1 (resistant:susceptible), obtained in the F_2_ population from the Jagger × TA5605 cross, suggested a monogenic dominant inheritance (Kuraparthy et al., 2007b). The authors failed to determine the chromosome location of the introgression using 23 5A-specific SSR markers. No signal from the alien segment was revealed by GISH, indicating that the introgressed segment is very small and cytologically undetectable. Polymorphic alleles between the resistant and susceptible bulks were revealed by the RFLP probe *KSUF11*, specific for group 2 of wheat. Eighteen additional 2BL-specific probes were further selected for characterizing the translocation in TA5605, and two of them, *KSUH16* and *BG123*, showed *Ae. triuncialis-*specific diagnostic polymorphism between resistant and susceptible bulks. Three probes co-segregated with leaf rust resistance in the F_2_ mapping population. The authors concluded that the translocation in TA5605 involved the group 2 chromosome of *Ae. triuncialis* and the 2L arm of wheat chromosome and occurred through homoeologous recombination (Kuraparthy et al., 2007b). The physical location of the translocation breakpoint was deduced considering the position of resistance-associated/not-associated RFLP markers on the genetic map. Translocation breakpoint in TA5605 was localized in the deletion bin 2L-0.89–1.00 of the consensus physical map, and the size of the introgressed segment was <10% of the long arm of wheat chromosome 2BL (Kuraparthy et al., 2007b). Genomic origin of *Ae. triuncialis* chromosome (2U^t^ or 2C^t^) involved in this cryptic translocation designated T2BS·2BL-2^t^L(0.95) remained unknown.

The leaf rust-resistant introgression line 2K-69–4 was produced by backcrossing CS5A(5U^t^) substitution line with CS *ph^I^* to induce heterologous chromosome pairing (Aghaee-Sarbarzeh et al., 2002). GISH revealed a homozygous translocation; the major part of the recombinant chromosome was from chromosome 5U^t,^ and only the distal part of the short arm corresponded to 5AS. *Aegilops triuncialis* accession #3549, DS5U^t^(5A), and the translocation line 2K-69–4 were all resistant to the most virulent in Indian leaf rust pathotypes 77A-1 and 77-2. Moderate susceptibility of DS5U^t^(5A) to leaf rust under field conditions (compared to high susceptibility of the recurrent wheat parent) suggested that chromosome 5U^t^ carried a gene conditioning slow rusting against the prevalent leaf rust races. Because the recombinant chromosome of 2K-69–4 consisted mainly of 5U^t^, which may bring many agronomically undesirable genes, this translocation cannot be directly used for wheat improvement.

To transfer the mildew resistance gene located on 5U^t^ (Harjit-Singh et al., 2000) and to reduce the size of the introgressed fragment, the DS 5U^t^(5A) line was crossed and backcrossed with Pavon *ph1bph1b* to obtain a hybrid homozygous for *ph1b* (Kamboj et al., 2020). The plants homozygous for *ph1b* and monosomic for 5U^t^/5A were selected using molecular markers and crossed with WL711 to induce 5U^t^-5A recombinants by homoeologous pairing. Homozygous recombinants were obtained after five rounds of selfing, and in each generation, only plants with square heads were propagated (“spelta” heads served as a marker for the absence of recombination, and plants were abounded). A total of 367 F_4_ substitution recombinant lines were screened for mildew resistance in the lab and under field conditions, and 68 F_5_ lines with varying incidence of powdery mildew (0–9 score) were genotyped using 29 wheat 5A-specific SSR markers. Of the 367 F_5_ lines, only eight were resistant to powdery mildew (two lines with spelt head and six with square head), while the remaining 259 were affected by the disease to varying degrees. The segregation ratio of the disease resistance presumed that it is governed by several genes. Analysis of 68 selected lines with different responses to infection using 18 transferable and polymorphic 5A-specific SSR markers allowed to identify three main regions of 5U^t^—one on 5U^t^S/5AS and the other two on 5U^t^L/5AL, associated with powdery mildew resistance (Kamboj et al., 2020). The major resistance gene was apparently localized in the short 5U^t^/5A arm. Meiotic analysis of four randomly selected mildew-resistant lines showed normal chromosome pairing (21″), indicating that the translocation of 5U^t^ fragments conferring mildew resistance into 5A was compensating (Kamboj et al., 2020). Noteworthy, the resistance gene(s), which was introgressed from *Ae. triuncialis* to wheat more than 25 years ago (Harjit-Singh et al., 2000), continued to maintain resistance against the diverse prevalence in Indian pathotypes of *Blumeria graminis* for over 18 years (Kamboj et al., 2020).

Introgressive DS 5U^t^(5A) line (BTC17) also served as donor of the extra-soft grain texture trait controlled by puroindolines (Sharma et al., 2020). A total of 367 5U-5A substitution recombinant lines developed by Kamboj et al. (2020) were screened for the presence of *Ae. triuncialis-*derived *Pina* and *Pinb*, but only 23 of them were found to contain both alleles (Sharma et al., 2020). Grain hardness index of recombinants with functional puroindolines was reduced from 6% to 67%. Using the 5A-specific SSR markers, the authors established that the 5U^t^ fragment containing puroindoline loci was translocated to the telomeric end of wheat chromosome 5AS, which was confirmed by sequencing of *Pina* and *Pinb* genes (Sharma et al., 2020). The obtained lines could be in demand in breeding soft wheat varieties for the cookie and biscuit industries. As puroindoline proteins extracted from soft-grain lines exhibited antimicrobial activity against gram-positive and gram-negative bacteria, they can be utilized in the pharmaceutical industry.

Two novel genes controlling leaf and stripe rust resistance were introduced from *Ae. triuncialis* accession # pau 3462 to the susceptible wheat cultivar WL711 (Arora et al., 2021). *Aegilops triuncialis* was crossed and backcrossed by CS *ph1b* to induce recombination between *Aegilops* and wheat chromosomes. BC_2_ progeny with the recurrent plant type and resistant to both pathogens was selected in subsequent generations to develop the stable, resistant BC_2_F_7_ plants phenotypically similar to WL711. Inheritance and chromosome location of resistance genes were studied in the F_2_ and F_2:3_ mapping populations obtained by crossing the stable introgression line IL tri with the wheat cultivar WL711. According to the segregation ratio of resistant/susceptible plants, both diseases are controlled by single dominant genes, temporarily designated *LrTri* and *YrTri*, which were transferred to wheat as independent fragments (Arora et al., 2021). Based on molecular-genetic analysis with 614 SSR markers to all wheat chromosomes, the *LrTri* was mapped on chromosome 7BS/7DS at a distance of 11.2 cM from the SSR marker *Xwmc606* (Arora et al., 2021), although the location of the yellow rust resistance gene remained unknown.

*Aegilops triuncialis* is highly resistant to cereal cyst nematode caused by *H. avenae* (Person-Dedryver et al., 1985; Benveniste-Levkovitz et al., 1993; Narang et al., 2019). To transfer this trait to wheat, Romero et al. (1998) obtained two vigorous male-sterile amphihaploids *T. turgidum* (H-1-1) × *Ae. triuncialis* (A-1), which were crossed with the bread wheat cultivar Almatense (H-10-15). The resulting hybrids were highly sterile, but produced eight seeds. The plants obtained from these seeds contained 28–41 chromosomes and were highly sterile. After eight rounds of selfing, 49 translocation lines (TR) with chromosome number 40 (95%) or 41 (5%) have been developed. The fertility of hybrids was still very low, but the surviving plants were vigorous. Low fertility was probably caused by the *Ph-*suppressing effect of the C^t^ genome (Romero et al., 1998) or by the action of Gc factors carried by *Ae. triuncialis* chromosomes 2C^t^ and 3C^t^ (Endo, 2007). *Gc* genes could also induce heterologous chromosome translocations in hybrids.

According to field tests, 9 of 49 TR lines were not infested with CCN pathotype Ha71, and one of these lines—TR-535—was chosen for further studies because of its good performance in all resistance tests. Segregation analysis of the BC_5–6_ families obtained from the TR-535 × *T. aestivum* cross showed that CCN resistance transferred from *Ae. triuncialis* is controlled by a single dominant gene tentatively designated *CreAet* (Romero et al., 1998), currently *Cre7* (Wheat Gene Catalogue, 2024). The authors suggested that CCN resistance in TR-535 was derived from the C^t^ genome (Romero et al., 1998); however, the chromosome location of the *Cre7* was not determined, and its genome origin still needs to be confirmed.

The same translocation line TR-353 (2*n*=41) and its euploid derivative TR-3531 (2*n*=42) also served as donors of Hessian fly resistance to wheat. Martin-Sanchez and co-authors (Martin-Sanchez et al., 2003) found that *Ae. triuncialis*-derived resistance is inherited as a single Mendelian factor, and the newly discovered gene designated *H30* is non-allelic to already known genes. The resistance was associated with the *Acph-U1* marker, which was also identified in *Ae. triuncialis*, *Ae. umbellulata*, and amphiploid CS-*Ae. umbellulata*, but was absent in wheat–*Ae. umbellulata* addition lines and in *Ae. caudata*. Based on these data, Martin-Sanchez et al. (2003) proposed that Hessian fly resistance was transferred from the U^t^ genome, and the *H30* gene is located on a segment of chromosome 4U^t^ translocated to chromosome 4D of wheat. Importantly, the authors were able to introduce resistance to the Hessian fly (less often, together with the oat nematode) to 16 elite breeding lines; i.e., their material became a valuable source of these traits for wheat improvement.

Gametocidal chromosomes of *Ae. triuncialis* were first discovered in 1975 by Endo and Twunewaki (Endo and Tsunewaki, 1975). They noticed that all wheat–*Ae. triuncialis* alloplasmic lines were characterized by extreme female sterility. All wheat lines with substituted cytoplasm from *Ae. triuncialis*, without any exceptions, carried additional acrocentric chromosome from their cytoplasm donor. The author supposed that sterility was caused by the unviability of gametes lacking the alien chromosome, which were named gametocidal or “gamete killing” (Endo, 2015; Said et al., 2024). Two gametocidal chromosomes, 2C^t^ and 3C^t^, were identified in *Ae. triuncialis*, which carried the *Gc1-C1* and *Gc3-C1* genes, respectively (Endo, 1979; Endo, 1996). Gc-chromosome 2C^t^ expressed mild, semilethal effects in all wheat varieties (Endo, 1982; Endo, 2007). Chromosomes 3C^t^ and its derivative 3C^SAT^ expressed different actions in CS background: that of 3C^t^ was lethal but that of 3C^SAT^ was semilethal (Endo TR, 1996). Structurally rearranged chromosomes of CS that emerged under the action of Gc factors of 2C^t^ and 3C^SAT^ were cytologically identified and isolated, giving rise to a series of wheat deletion stocks (Endo TR, 1996), as well as to deletion stocks of rye (Masoudi-Nejad et al., 2002; Li et al., 2013; Endo, 2021) and barley (*H. vulgare* and *H. chilense*) chromosomes added to CS (Serizawa et al., 2001; Nasuda et al., 2005; Said and Cabrera, 2009; Sakai et al., 2009). Due to their ability to induce spontaneous chromosome breaks, Gc chromosomes were employed for reducing the size of alien introgressions. The ability of *Ae. triuncialis* cytoplasm to induce cytoplasmic male sterility could find practical application in hybrid wheat breeding (Tsunewaki et al., 1974).

Currently, no reports on the practical utilization of the genetic material of *Ae. columnaris* (**Figure 1E**) have been reported, although this species possesses a number of valuable traits, including resistance to diseases and different abiotic factors (**Table 3**). Three primary amphidiploids were produced in the All-Russian Research Institute of South-East, Saratov (ARISER) by crossing three common wheat cultivars, Dobrynya, Saratovskaya-68, and L-503 with *Ae. columnaris* (acc. k-1193). Hybrid seeds were well-developed and viable, but the F_1_ plants were sterile and were backcrossed to the recurrent wheat cultivars. The BC_1_ and BC_2_ progenies were either backcrossed again to wheat or self-pollinated. As a result, 57 introgressive lines with different substitutions and additions have been developed (Badaeva et al., 2018). According to preliminary observations, introgression of chromosome 5X^c^ was associated with leaf rust resistance in hot and dry conditions of the middle Volga region (Badaeva et al., 2018). Some substitutions were associated with enhanced salt tolerance (Dashtoian et al., 2023). The cytoplasm of this species induces cytoplasmic male sterility in wheat (Tsunewaki et al., 1974).

We failed to find any publications on the transfer of useful traits from hexaploid *Ae. juvenalis* (**Figure 1C**), although all accessions of this species are immune to powdery mildew (Valkoun et al., 1985; Boguslavsky and Golik, 2003) and highly resistant to *Fusarium* root rot (Boguslavsky and Golik, 2003). A relatively recent article reported the production of a decaploid hybrid *Ae. juvenalis* × durum wheat (Takumi et al., 2020), and hybrid and several introgressive lines of *Ae. juvenalis* with triticale have been developed, although not characterized further (Gradzielewska et al., 2012).

## Conclusion

Summarizing the above information, *Aegilops* species containing the U genome (**Table 1**) have already contributed many valuable genes, especially disease resistance genes, some of which have found a broad application in common wheat breeding (**Table 2**). Thus, over 79 commercial wheat cultivars, predominantly from the US, possess the *Ae. umbellulata-*derived gene *Lr9.* Many other genes conferring leaf rust (*Lr54*, *Lr76*), stem rust (*Sr53*), yellow rust (*Yr37*, *Yr40*), powdery mildew (*Pm9*, *Pm29*), and nematodes (*CreX*, *CreY*, *Cre7*) have also been introgressed from species of the U genome group into wheat, although they have not been used as extensively as *Lr9.* All relevant publications demonstrate that *Aegilops* species carrying the U genome constitute a unique and highly promising genetic resource for improving cultivated wheat, not only with regard to disease resistance but also to other agronomically important traits. They can serve and have already been used as donors of draught, heat and salt tolerance, high micronutrient content, and high baking quality. The mechanisms underlying these traits, however, remain not fully understood and need further investigation. Detailed examination of the gene pool of *Aegilops* species, including the U genome group, holds a great promise for developing wheat varieties of the next generation that combine high productivity with multifaceted resistance to both biotic and abiotic stresses. There is no doubt that the genetic potential of the U genome group of *Aegilops* is far from exhausted and they can become donors of many new important traits (**Table 3**) and expand the genetic diversity of durum and common wheat. In summary, the U genome-bearing *Aegilops* species represent a unique genetic reservoir whose significance will only grow with the development of modern breeding technologies.
